# Traceless Cleavage of Protein–Biotin Conjugates under Biologically Compatible Conditions

**DOI:** 10.1002/cbic.201700214

**Published:** 2017-07-19

**Authors:** Joseph Cowell, Matthew Buck, Ali H. Essa, Rebecca Clarke, Waldemar Vollmer, Daniela Vollmer, Catharien M. Hilkens, John D. Isaacs, Michael J. Hall, Joe Gray

**Affiliations:** ^1^ Institute for Cell and Molecular Biosciences Newcastle University Newcastle upon Tyne NE2 4HH UK; ^2^ School of Chemistry Newcastle University Newcastle upon Tyne NE2 7RU UK; ^3^ Musculoskeletal Research Group Institute of Cellular Medicine Newcastle University Newcastle upon Tyne NE2 4HH UK; ^4^ Department of Chemistry College of Science University of Basrah Basrah Iraq

**Keywords:** affinity purification, protein modifications, proteomics, reversible biotinylation, traceless cleavage

## Abstract

Biotinylation of amines is widely used to conjugate biomolecules, but either the resulting label is non‐removable or its removal leaves a tag on the molecule of interest, thus affecting downstream processes. We present here a set of reagents (RevAmines) that allow traceless, reversible biotinylation under biologically compatible, mild conditions. Release following avidin‐based capture is achieved through the cleavage of a (2‐(alkylsulfonyl)ethyl) carbamate linker under mild conditions (200 mm ammonium bicarbonate, pH 8, 16–24 h, room temperature) that regenerates the unmodified amine. The capture and release of biotinylated proteins and peptides from neutravidin, fluorescent labelling through reversible biotinylation at the cell surface and the selective enrichment of proteins from bacterial periplasm are demonstrated. The tags are easily prepared, stable and offer the potential for future application in proteomics, activity‐based protein profiling, affinity chromatography and bio‐molecule tagging and purification.

The ability to selectively enrich tagged biomolecules from a complex mixture has had a wide‐ranging impact in biology.^1^ Biotinylation of both engineered and wild‐type proteins enables the use of avidin–biotin affinity techniques based on the formation of a strong noncovalent biotin–avidin complex (*K*_d_≈10^−14^–10^−15^ mol L^−1^).^2^ Common applications include purification with solid‐supported avidins and labelling through solution‐phase avidin–biotin complexation. However, disruption of these complexes (e.g., to allow protein elution) typically necessitates the use of harsh conditions that are not compatible with maintaining protein function.^3^ Elution of biotin conjugates from solid‐supported avidins can be improved by reducing the *K*_d_ of the complex through the use of either biotin analogues^4^, ^5^ or mutant avidins.^6^ Alternatively the use of cleavable biotinylation reagents allows selective release of the protein through proteolytic, photolytic or chemical cleavage.^7^ Chemically cleavable linkers include diazobenzenes (cleavage: sodium dithionite),^8^ vicinal diols (sodium periodate),^9^ bisarylhydrazones (catalytic transamination),^10^ acylhydrazones,^11^ silyl ethers,^12^ maleic anhydrides^13^ and acetals (acid),^14^ levulinoyl esters (hydrazine),^15^ and dithanes,^16^ bromomaleimides^17^ and conjugate acceptors^18^ (thiol). However, upon cleavage, residual atoms from the linker remain attached to the protein, potentially complicating downstream processes such as activity assays or proteomic analysis. Thus, traceless cleavage^13^, ^17^, ^18^ in which the unmodified parent protein is released is a key requirement for new biotinylation reagents.

Herein we describe the synthesis of sulfonyl‐based reversible amine (or “RevAmine”) biotinylation reagents and demonstrate how they are capable of facile functionalization of proteinaceous amines with subsequent traceless cleavage triggered under biologically compatible, basic conditions (pH≈8). Sulfonyl‐triggered elimination reactions have been reported (e.g., amine protection,^19^ protein–amine20a,20b and polypeptide–oligonucleotide^21^ crosslinking, polypeptide synthesis^22^ and the slow release of drug molecules^23^), but not under biologically compatible conditions. Therefore sulfonyl‐triggered eliminations have yet to find wider application in biology and more specifically in reversible biotinylation chemistry.

Our RevAmine reagents contain a bioconjugatable *N*‐hydroxysuccinimide activated‐carbonate and form a (2‐(alkylsulfonyl)ethyl) carbamate linker that is stable at neutral and acidic pH, but capable of traceless cleavage via an elimination/decarboxylation cascade in the presence of weak base (Scheme 1). Our first‐generation tagging reagent **4** incorporates the desired bioconjugation/release motif and is constructed from simple starting materials. 1,3‐Dicyclohexylcarbodiimide (DCC) coupling of (+)‐biotin (**1**) with 2,2′‐sulfonylbis(ethan‐1‐ol) (**2**), to give the desired ester **3** is followed by treatment with bis(2,5‐dioxopyrrolidin‐1‐yl) carbonate to give **4** (Scheme 2, Figures S1 and S2 in the Supporting Information).

**Scheme 1:**
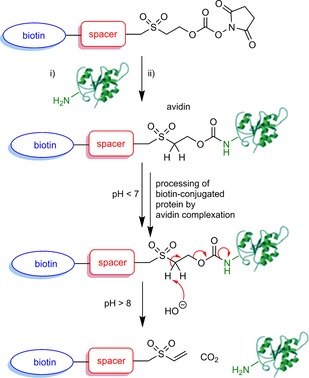
RevAmine biotinylation reagents (i), which form a (2‐(alkylsulfonyl)ethyl) carbamate upon bioconjugation (ii), allow sulfonyl‐triggered, biocompatible traceless release.

**Scheme 2:**
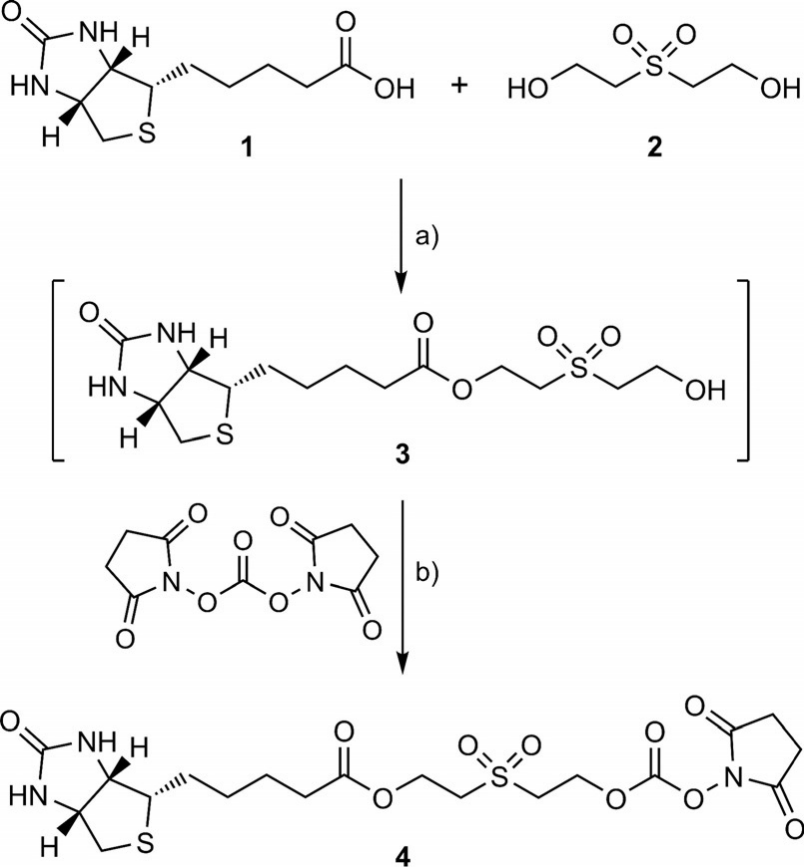
Synthesis of RevAmine reagent **4**. a) 4‐(Dimethylamino)pyridine (DMAP), DCC, CH_2_Cl_2_, RT, 9 days; b) pyridine, MeCN, RT, 30 min.

We then investigated the bioconjugation of both the decapeptide CD31 (665–674): HNDDVRNHAM and the HA antigen peptide: YPYDVPDYA (**4**:peptide 2:1, 2 h, room temperature, 100 mm sodium phosphate, pH 7.4). Analytical HPLC and MS showed that a single molecule of **4** had been incorporated into each molecule of CD31 (665–674) peptide (MALDI: *m*/*z* 1614.61 [*M*+H]^+^, Figure S5); electrospray MS/MS analysis confirmed that biotinylation had occurred at the N terminus.

The CD31 (665–674)–**4** conjugate was isolated by HPLC, dried, reconstituted in water and treated with basic aqueous ammonia solution (dilutions down to 0.01 %). HPLC/MALDI analysis showed it to display the desired behaviour of rapid, traceless cleavage to regenerate the unmodified peptide (Figure S5). More significantly, traceless cleavage of peptide–**4** conjugates was also achieved under very mild conditions (100 mm ammonium bicarbonate, pH 8, 16 h, room temperature), as confirmed by HPLC and MS analysis (Figure S6, HA antigen peptide example). To evaluate a biocompatible avidin capture/release strategy, HA peptide–**4** conjugate was bound to NeutrAvidin beads (PBS, 1 h, room temperature), washed and then eluted overnight (100 mm ammonium bicarbonate, pH 8, 16 h, room temperature). Successful cleavage of the HA peptide–biotin linker allowed hitherto unreported, clean elution of unmodified HA peptide under extremely mild, biocompatible conditions (Figure S7).

Bovine serum albumin (BSA) was chosen as a test substrate to evaluate the potential for protein biotinylation. The reaction of BSA with **4** (**4**:BSA 20:1, PBS, 1 h, room temperature) was examined by MALDI, which revealed that approximately eight biotin groups were incorporated per BSA (Figure S8). Biotinylated BSA was captured by incubation with neutravidin beads. No BSA release was observed by PAGE during elution with PBS (pH 7.4), whereas elution of regenerated, unmodified BSA occurred under a range of mild conditions (100 mm ammonium bicarbonate, pH 8.0, 8.5, or 9.0 as well as 0.1 % ammonium hydroxide; Figure S9).

This facile conjugation and removal of biotin, suggested that **4** could be used in the investigation of more complex biological problems, such as in cell‐surface analysis. Therefore we carried out a labelling experiment that would allow a visual demonstration of cell‐surface biotinylation. Jurkat cells were sequentially incubated with **4** (PBS, 1 h), streptavidin/Alexa Fluor 568 conjugate and counterstained with 4′,6‐diamidino‐2‐phenylindole (DAPI). Red fluorescence (Figure 1, left) showed that **4** had been incorporated onto the cell surface, presumably through reaction with cell‐surface amines. Similarly prepared Jurkat cells that were additionally exposed to 1 % aqueous ammonia (15 min) showed greatly diminished surface labelling. This demonstrates that traceless cleavage of the biotin tag is possible without noticeable cell disruption (Figure 1, right).

**Figure 1:**
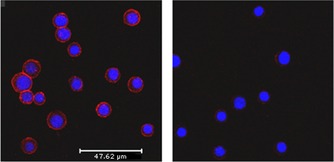
Confocal fluorescent imaging of surface‐biotinylated Jurkat cells (red: streptavidin/Alexa Fluor 568 conjugate, blue: DAPI). Left: Jurkat cells labelled with **4**, DAPI and streptavidin/Alexa Fluor 568. Right: Jurkat cells labelled with **4**, but pretreated with 1 % ammonia solution for 15 min prior to labelling with streptavidin/Alexa Fluor 568 and DAPI.

Building on the success of **4** as a tracelessly cleavable biotinylation reagent, we tried to create a second‐generation reagent that maintained the desired functionality but with improved synthetic accessibility. Bis(2,5‐dioxopyrrolidin‐1‐yl) (sulfonylbis(ethane‐2,1‐diyl)) bis(carbonate) (BSOCOES, **5**) was treated in a 1:1 ratio with (+)‐biotin hydrazide **6** (DMSO, 1 h, room temperature) to give RevAmine tag **7** in a single synthetic step (Scheme 3, Figures S3 and S4). Following purification, **7** could be stored in dry DMSO for several months at −20 °C without any noticeable loss of reactivity. Tests showed that **7** exhibited comparable bioconjugation and traceless‐cleavage chemistry to RevAmine tag **4** (Figures S5 and S10).

**Scheme 3:**
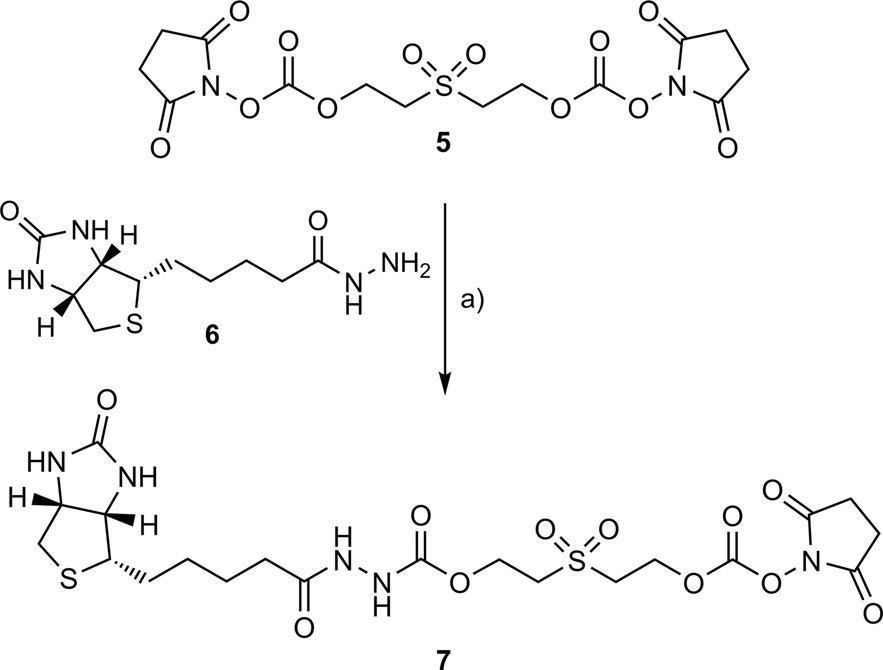
One‐step synthesis of RevAmine tag **7**. a) DMSO, RT, 1 h.

It should be noted that during prolonged tag‐cleavage of both **4** and **7** from test peptides, meditated by ammonium bicarbonate, we observed the formation of several minor by‐products (Figure S10, centre). MS analysis (Figure S6) suggested that these had arisen from the formation during tag cleavage of by‐product vinyl‐sulfones, which could possibly undergo subsequent nonselective Michael addition chemistry with the released peptide. The addition of dithiothreitol (DTT), as an in situ nucleophilic scavenger of the vinyl‐sulfone, resulted in a significant reduction in by‐product formation (Figure S10, top) and is recommended when performing cleavage experiments.20a, 24 The usefulness of tag **7** was then demonstrated in a series of tagging, avidin capture and biocompatible release experiments (in the presence of DTT) with three small protein targets, nuclease B (*Bacillus licheniformis*), cytochrome *c* (bovine) and apomyoglobin (equine). Tagging (tenfold excess of **7** over protein, 1 h, room temperature) showed a degree of substrate dependence, with incorporation of up to five biotin moieties under these conditions. The desalted, biotinylated proteins were captured on avidin beads, and the unmodified proteins were recovered by using ammonium bicarbonate (pH 8, 24 h, room temperature). HRMS measurements of all three eluted proteins gave excellent agreement with both the measured and predicted unmodified masses (Figures S11—S13).

As a final exemplar of the use of **7**, we decided upon a proteomic investigation of cell‐wall‐associated proteins in *Escherichia coli*.^25^ Porous outer‐membrane mutant *E. coli* cells (*amiABC*) were treated with **7**, followed by cell‐lysis and incubation with neutravidin beads to capture biotinylated proteins. Unbound proteins were removed by washing the beads with lysate buffer, then PBS (pH 7.4). Treatment with 100 mm ammonium bicarbonate, pH 9, overnight at 4 °C, triggered traceless linker cleavage, thus allowing elution of the previously bound, biotinylated proteins. The protein eluate was then subjected to trypsin digestion and proteomic analysis by LC/MS/MS and identification with Mascot software (Matrix Science, London). Due to the traceless nature of the cleavage reaction, no residual mass modifications in the software search were necessary to allow peptide identification^26^ (Tables S1 and S2). After subtraction of nonspecific IDs, the 64 proteins identified in the surface‐tagging experiment (Table S3) were submitted to a PANTHER over‐representation test search (http://pantherdb.org/webservices/go/overrep.jsp)^27^ against an *E. coli* background reference data set, with “cellular component” as the selected enrichment term. Of the proteins remaining following subtraction, the highest‐scoring enriched gene ontology terms shared by the genes on our list compared to the background distribution of annotations were: “*outer membrane‐bounded periplasmic space*”, “*external encapsulating structure*” and “*cell envelope*”. A more than fivefold selective enrichment of proteins from the periplasm had taken place (Figure S14).

In summary, we have outlined the basis for a series of biocompatible RevAmine tags that are synthetically accessible and are stable under neutral and acidic conditions, but that undergo traceless cleavage to regenerate the parent amine in the presence of a weak base. We have shown that RevAmine‐tagged proteins and peptides can undergo facile base‐mediated capture–release affinity purification on neutravidin solid supports and we have demonstrated the utility of such reagents in cell‐surface labelling and in proteomic analysis. Owing to their biocompatible traceless‐cleavage chemistry, we envisage numerous applications of RevAmine reagents in areas such as proteomics, activity‐based protein profiling, modifiable affinity chromatography supports, biomolecule tagging and biomolecule purification.

## Conflict of interest

*The authors declare no conflict of interest*

## Supporting information

As a service to our authors and readers, this journal provides supporting information supplied by the authors. Such materials are peer reviewed and may be re‐organized for online delivery, but are not copy‐edited or typeset. Technical support issues arising from supporting information (other than missing files) should be addressed to the authors.

Supplementary
