# Supplementary material for: Traceless Cleavage of Protein–Biotin Conjugates under Biologically Compatible Conditions
Source: Chembiochem. 2017 Jul 19;18(17):1688–91. doi: 10.1002/cbic.201700214 (PMC5708275; doi:10.1002/cbic.201700214)
Supplement: Supplementary file 1 — Supplementary [file CBIC-18-1688-s001.pdf]

CHEMBIOCHEM

Supporting Information

Traceless Cleavage of Protein–Biotin Conjugates under Biologically Compatible Conditions

Joseph Cowell,[b] Matthew Buck,[c] Ali H. Essa,[b, d] Rebecca Clarke,[b] Waldemar Vollmer,[a] Daniela Vollmer,[a] Catharien M. Hilkens,[c] John D. Isaacs,[c] Michael J. Hall,*[b] and Joe Gray*[a]

cbic_201700214_sm_miscellaneous_information.pdf

Supporting Figures and Tables

Figure S1 S2
Figure S2 S2
Figure S3 S3
Figure S4 S3
Figure S5 S4
Figure S6 S5
Figure S7 S6
Figure S8 S7
Figure S9 S7
Figure S10 S8
Figure S11 S9
Figure S12 S10
Figure S13 S11
Figure S14 S12
Table ST1 S13
Table ST2 S19
Table ST3 S24

General experimental procedures

Materials

Synthetic procedures S26

4: 2-((2-((((2,5-dioxopyrrolidin-1-yl)oxy)carbonyl)oxy)ethyl)sulfonyl)ethyl 5-((3a*S*,4*S*,6a*R*)-2-oxohexahydro-1*H*-thieno[3,4-*d*]imidazol-4-yl)pentanoate S26

7: 2-((2-((((2,5-dioxopyrrolidin-1-yl)oxy)carbonyl)oxy)ethyl)sulfonyl)ethyl 2-(5-((3a*S*,4*S*,6a*R*)-2-oxohexahydro-1*H*-thieno[3,4-*d*]imidazol-4-yl)pentanoyl)hydrazine-1-carboxylate S26

RevAmine peptide coupling S26

Analytical procedures

Analytical RP-HPLC S27
Base cleavage of RevAmine-modified peptides S27
MALDI MS Analysis of peptides S27
Infusion electrospray MS analysis S27
Modification of bovine serum albumin (BSA) with RevAmine 4 S27
MALDI MS analysis of BSA S27
Neutravidin capture and release of RevAmine 4-modified hemagglutinin antigen (HA) peptide S28
Neutravidin capture and release of biotinylated BSA with PAGE analysis S28
Fluorescence confocal microscopy of biotin RevAmine 4-labelled cells S28
RevAmine 7 protein modification, neutravidin capture and release S29
Trapping LC-MS analysis S29
E. coli surface labelling experiment S30
Nanoelectrospray MS/MS analysis S30
Proteomic data analysis S31

Figures and Tables

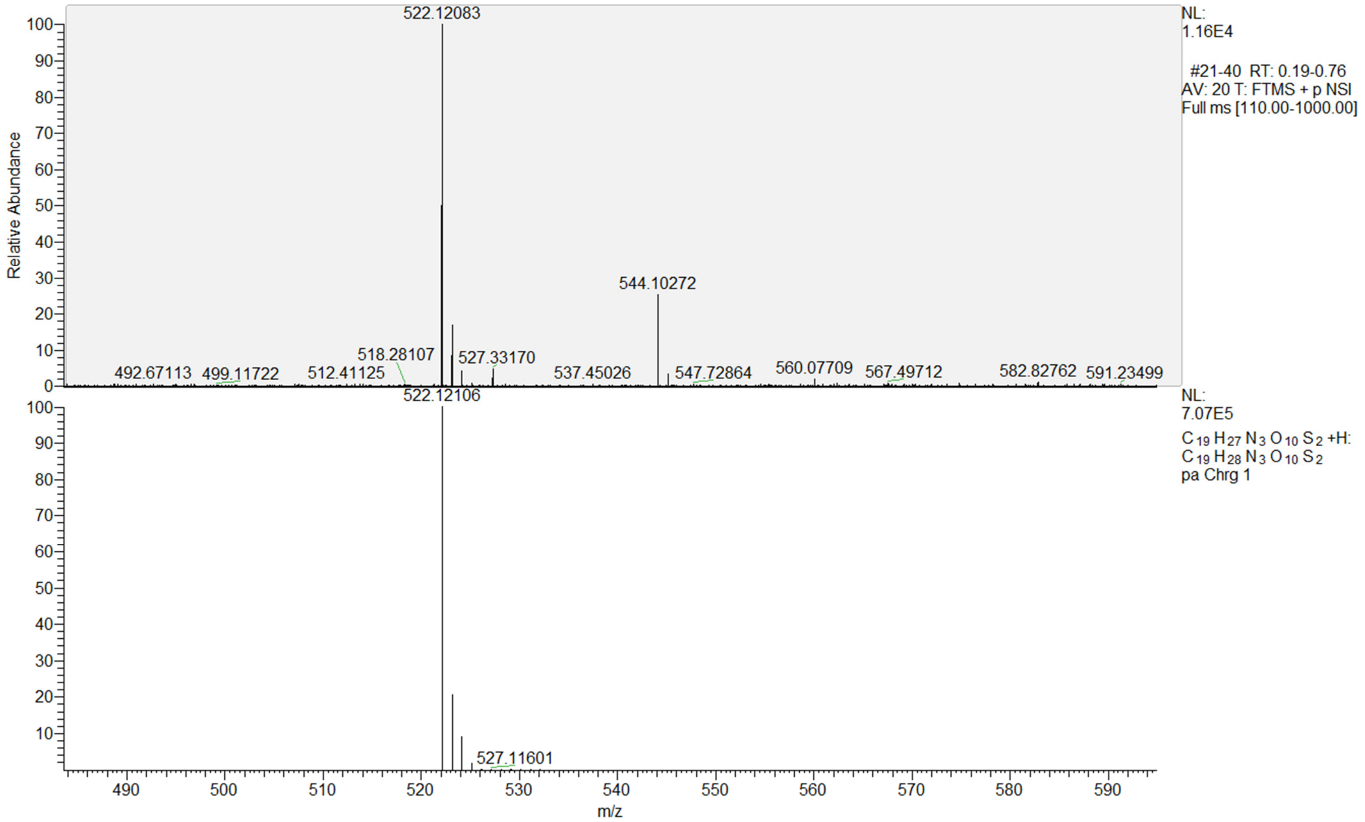

Figure S1. High resolution FT-MS confirming the identity of RevAmine **4**. The upper panel contains the actual MS spectrum, $[M+H]^{+}$ = 522.12083, whilst the lower panel is an isotope simulation based upon the correct formula.

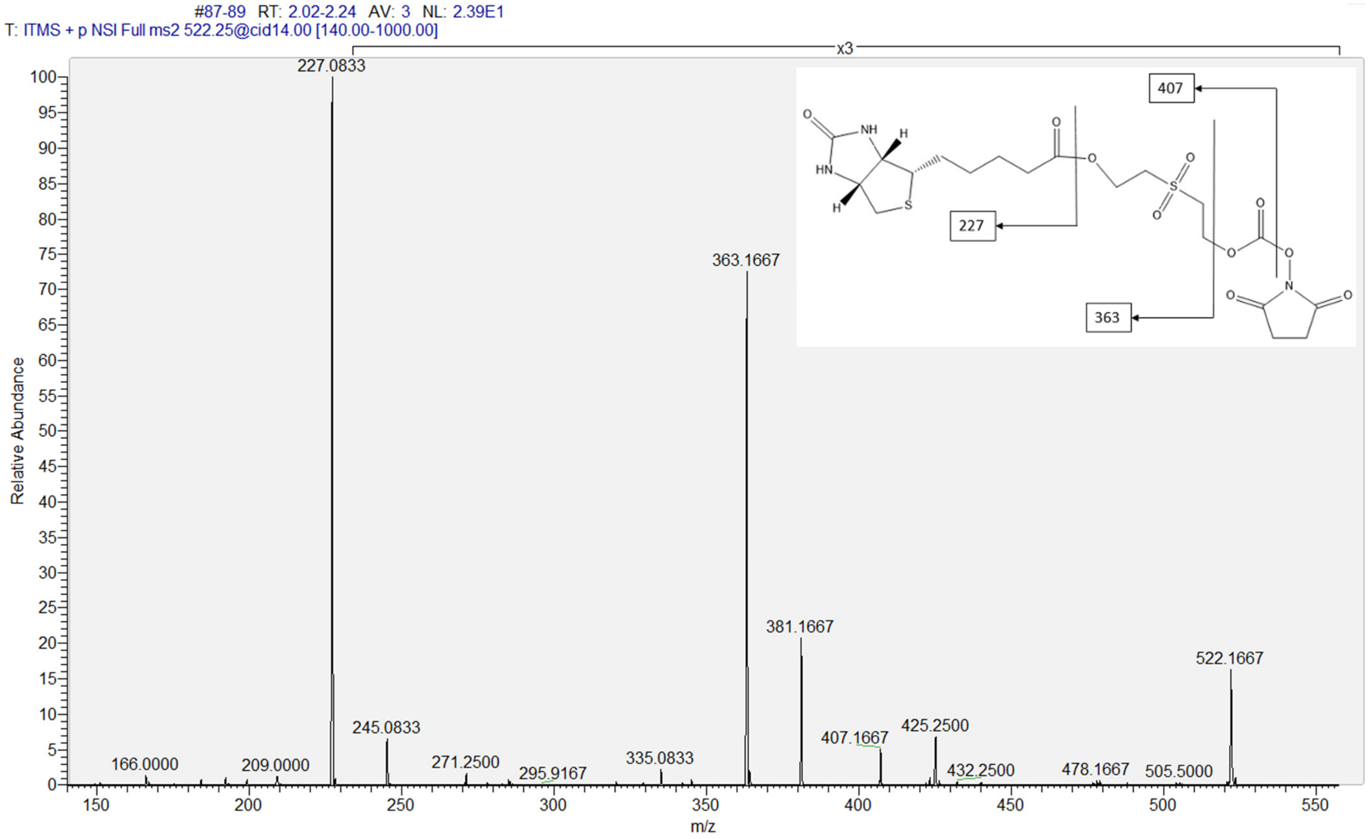

Figure S2. Additional MS/MS data confirming the identity of RevAmine **4**. The inset (top right) shows the structure with mass-labelled (nominal mass) fragmentations of observed ions in the MS/MS spectrum.

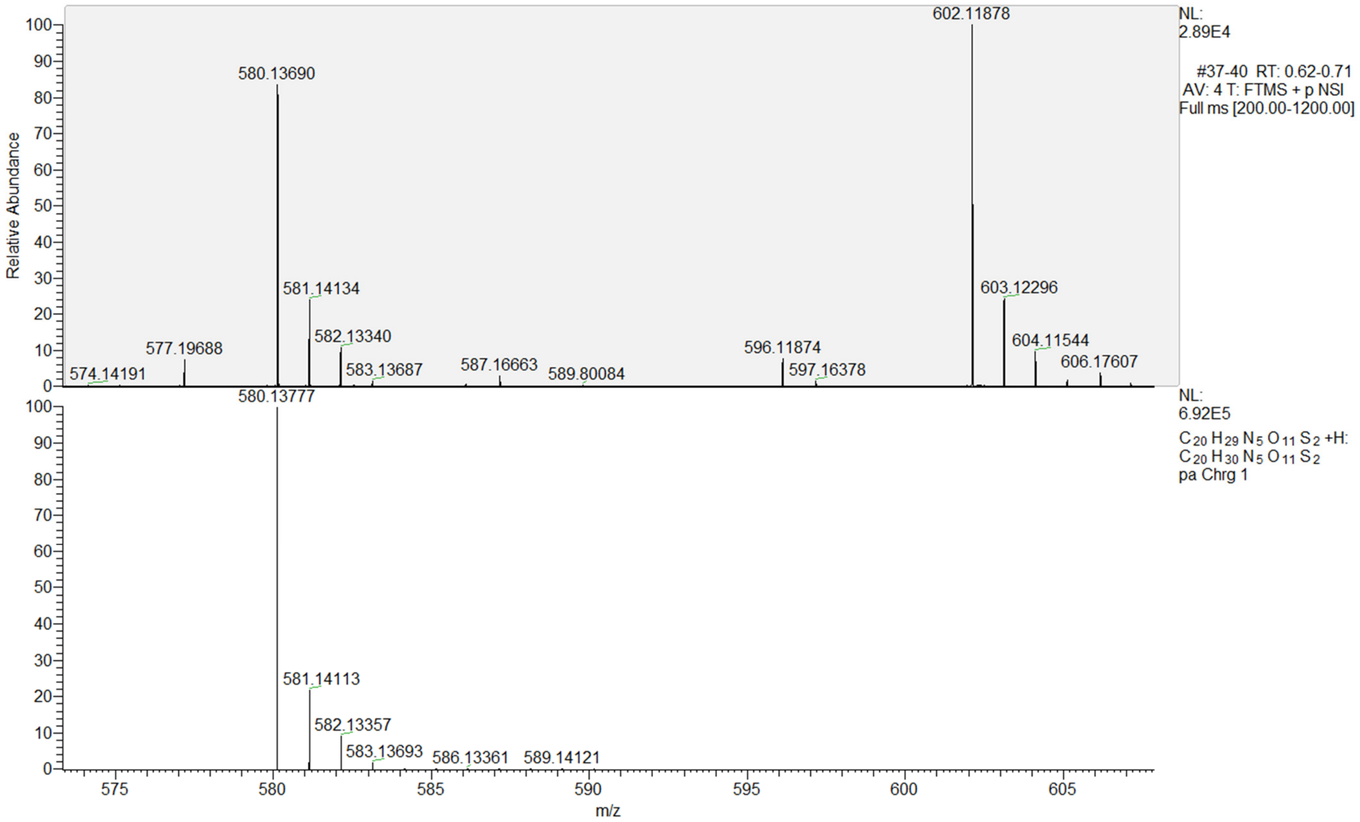

Figure S3. High resolution FT-MS confirming the identity of RevAmine **7**. The upper panel contains the actual MS spectrum, $[M+H]^+$ = 580.13690, whilst the lower panel is an isotope simulation based upon the correct formula.

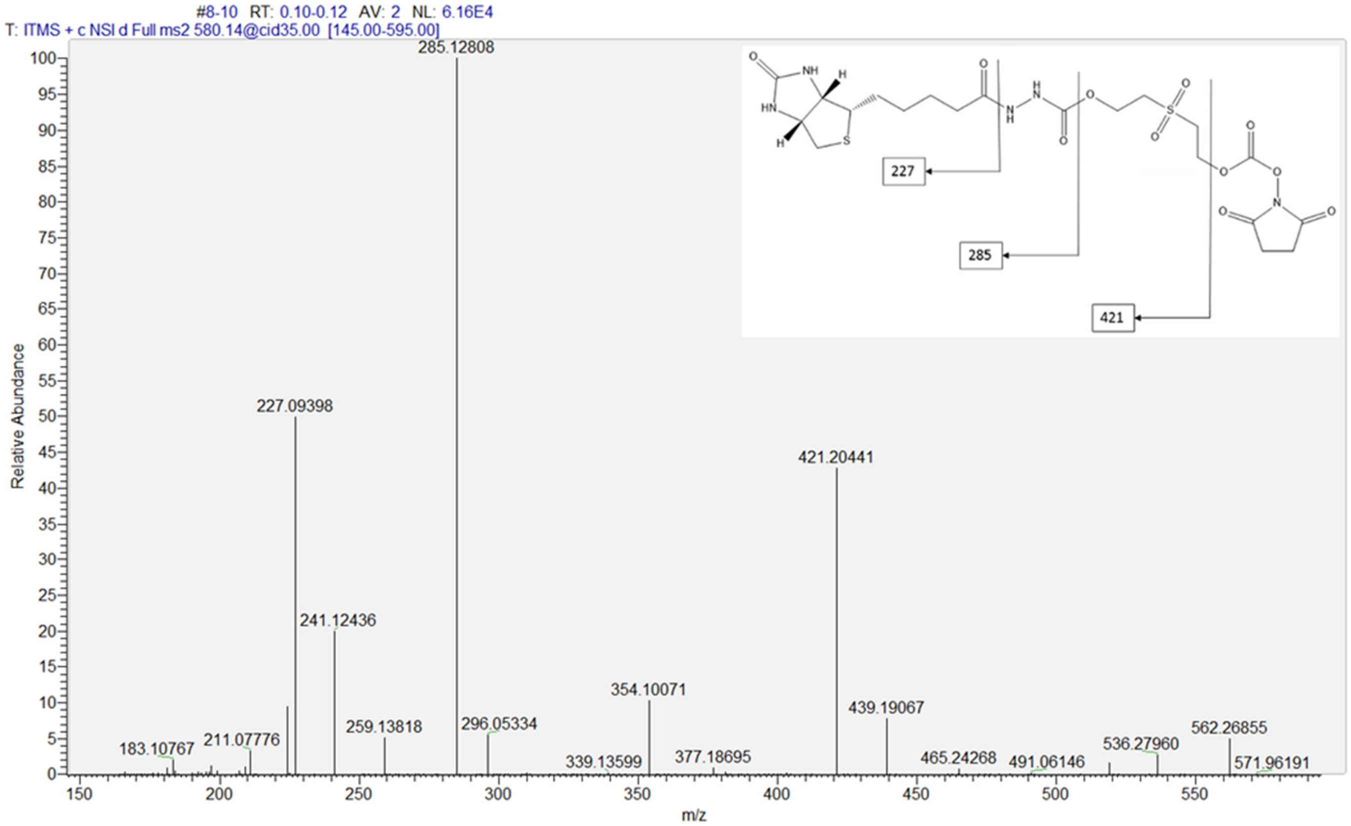

Figure S4. Additional MS/MS data confirming the identity of RevAmine **7**. The inset (top right) shows the structure with mass-labelled (nominal mass) fragmentations of observed ions in the MS/MS spectrum.

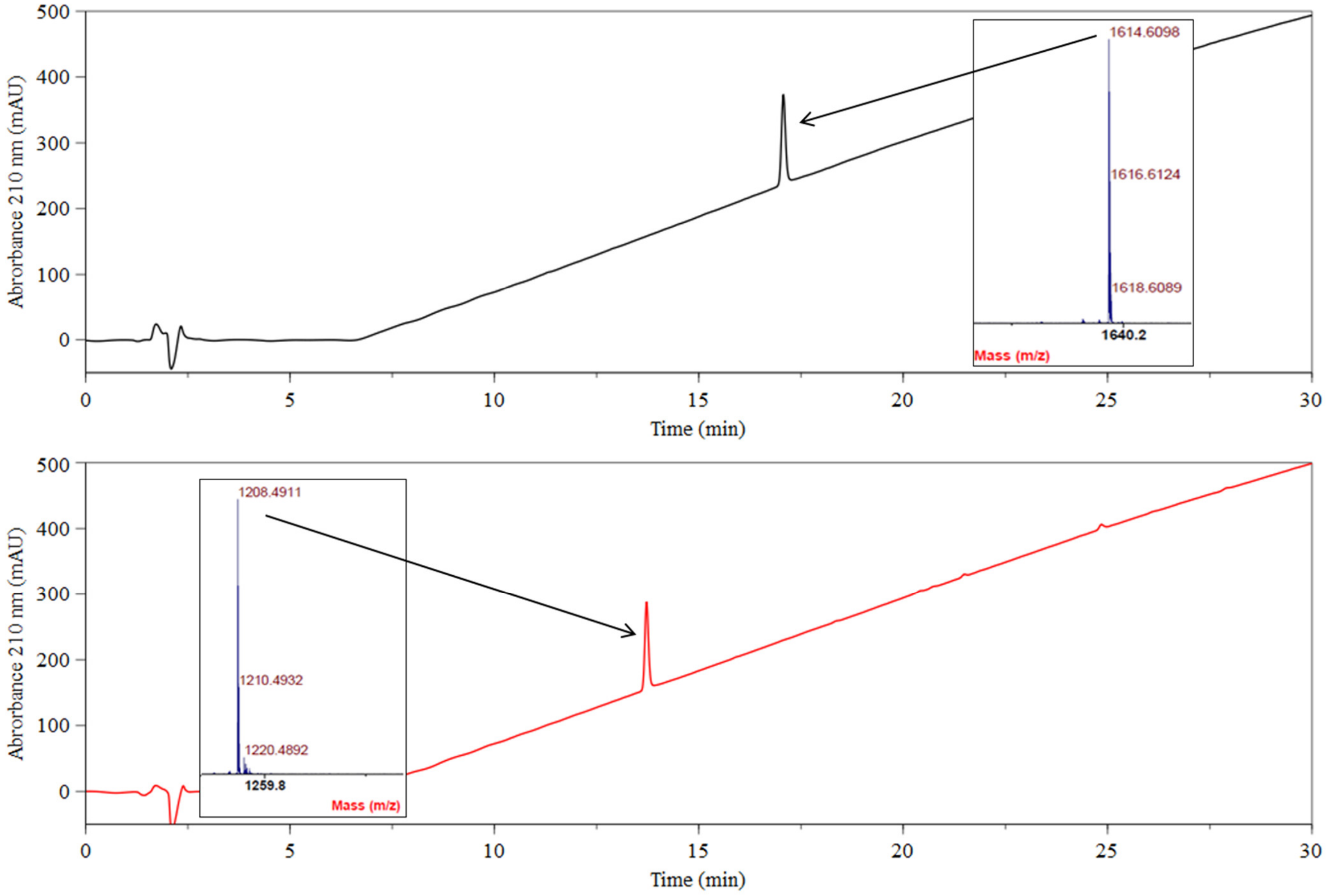

Figure S5. RP-HPLC analysis of CD31 (665-674)-**4** conjugate before (black) and after (red) treatment with 0.1% NH_4OH(aq), 0.5h @ RT. Insets show MALDI-MS analyses confirming peak identity and the traceless regeneration of the parent peptide $[M+H]^+$ = 1208.49.

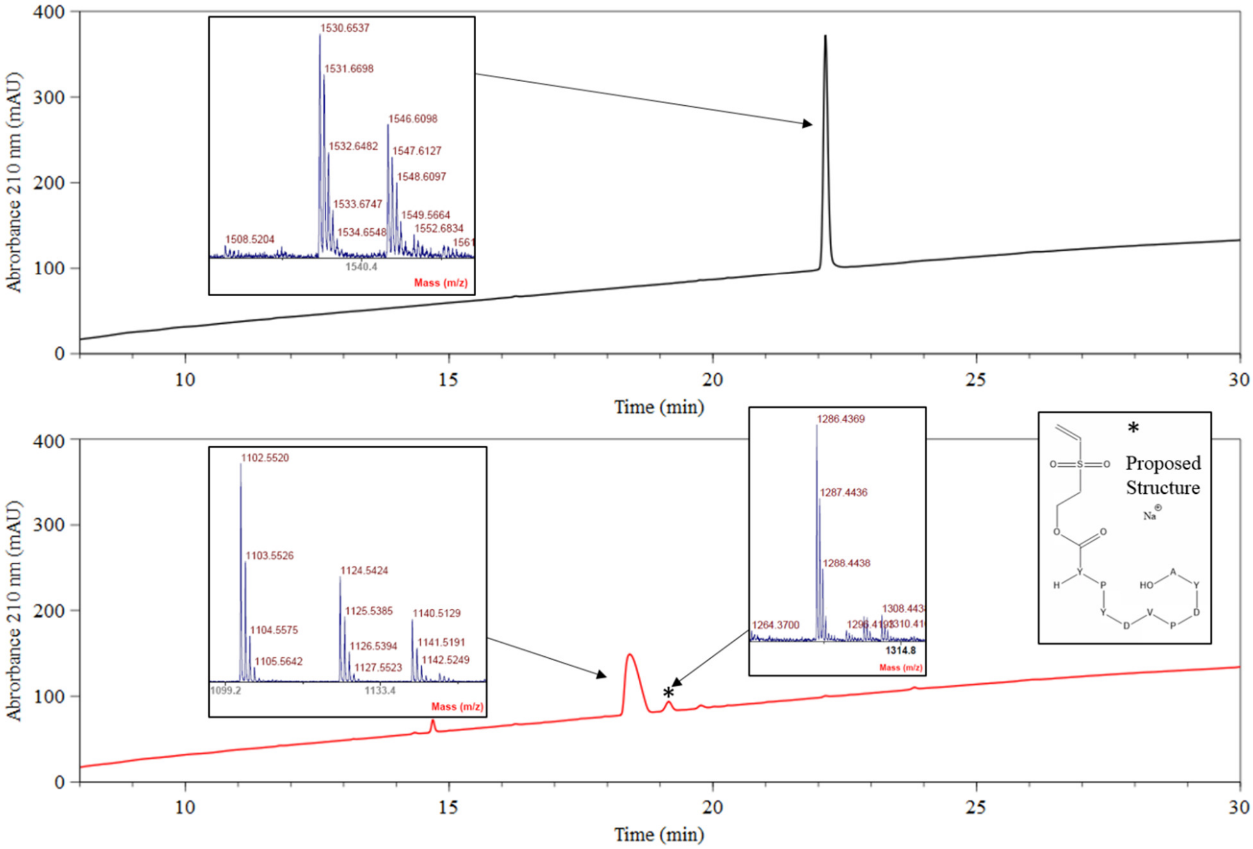

Figure S6. RP-HPLC analysis of RevAmine **4** modified HA peptide before (black) and after (red) treatment with 100 mM ammonium bicarbonate (aq), pH 8, overnight @ RT. Figure includes MALDI-MS insets confirming peak identity and the traceless regeneration of the parent peptide. The peak marked * is a residual vinyl sulphone cleavage intermediate (m/z=1286.44) with its proposed structure in the lower right inset.

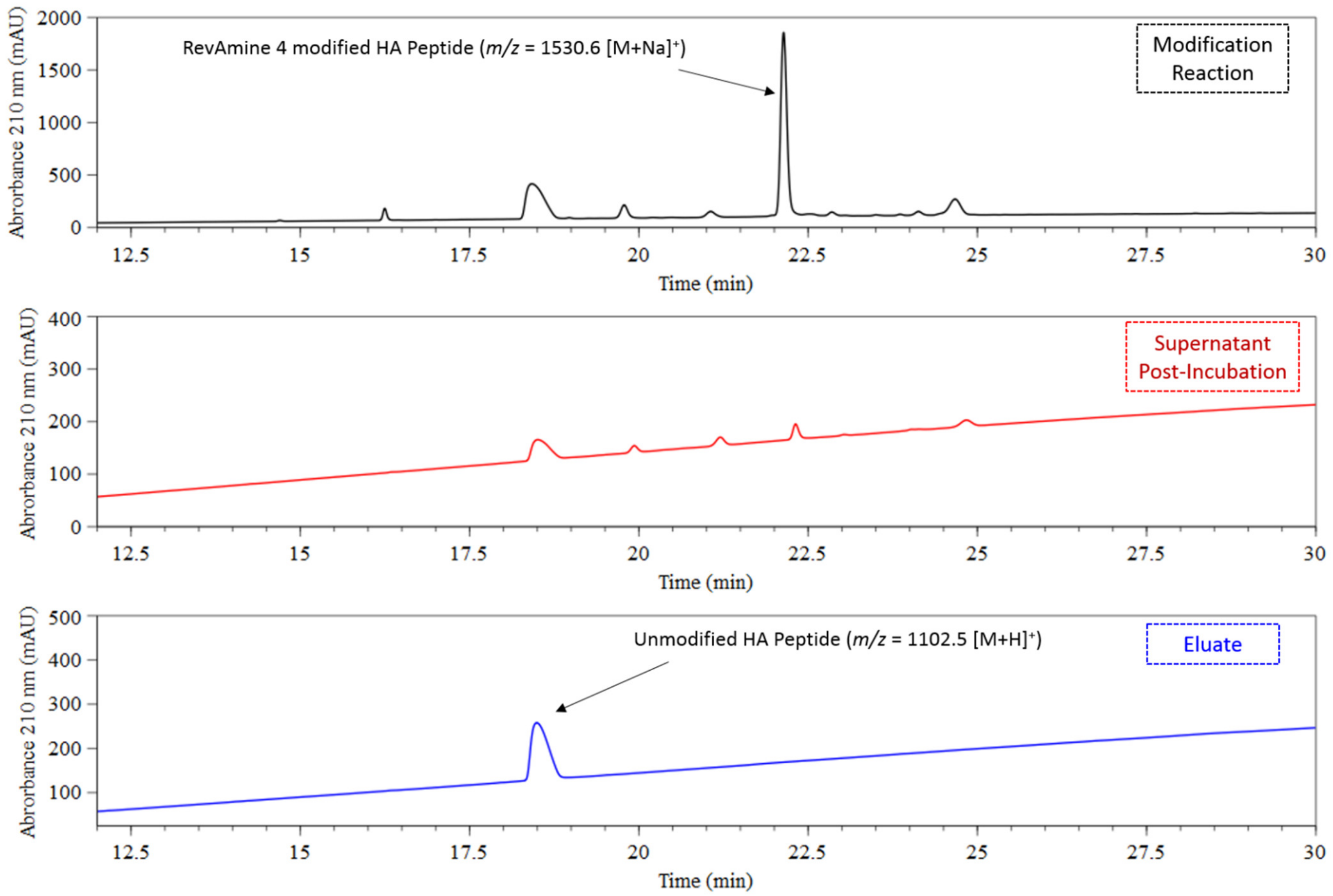

Figure S7. Biocompatible avidin-biotin capture-release. RP-HPLC analysis of RevAmine **4** modified HA peptide captured using neutravidin and released via overnight treatment with 100mM ammonium bicarbonate, pH 8 treatment. Top trace (black) represents the crude reaction mixture before application to immobilised neutravidin. Centre trace 2 (red) a sample of the reaction mixture after incubation with neutravidin beads (1 h) at RT showing almost complete removal of biotin-modified peptide. Bottom trace 3 (blue) a sample of the eluate after overnight incubation, pH 8, RT demonstrating clean, traceless release of unmodified HA peptide.

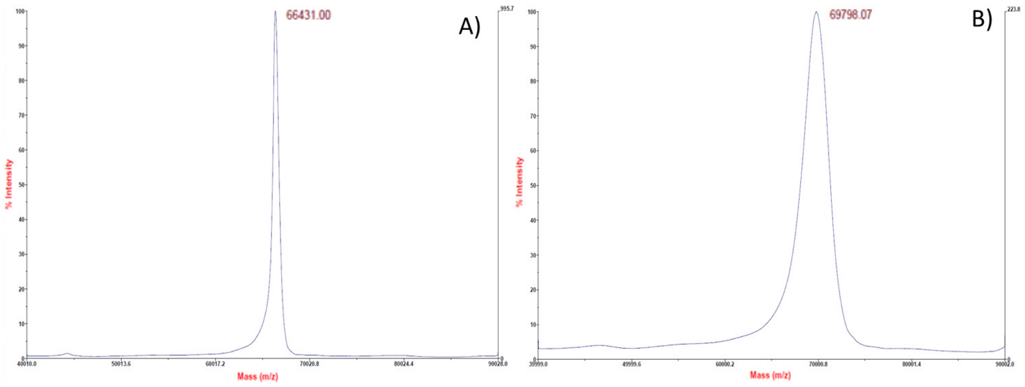

Figure S8. MALDI-TOF analysis of RevAmine **4**-modified BSA. A) Before modification ($[M+H]^+$ = 66431.0) , B) After modification ($[M+H]^+$ = 69798.0). The mass increase of 3367 Da corresponds to an average incorporation of approximately 8 modifications per molecule of BSA (modification mass = 406.47 (avg. mass)).

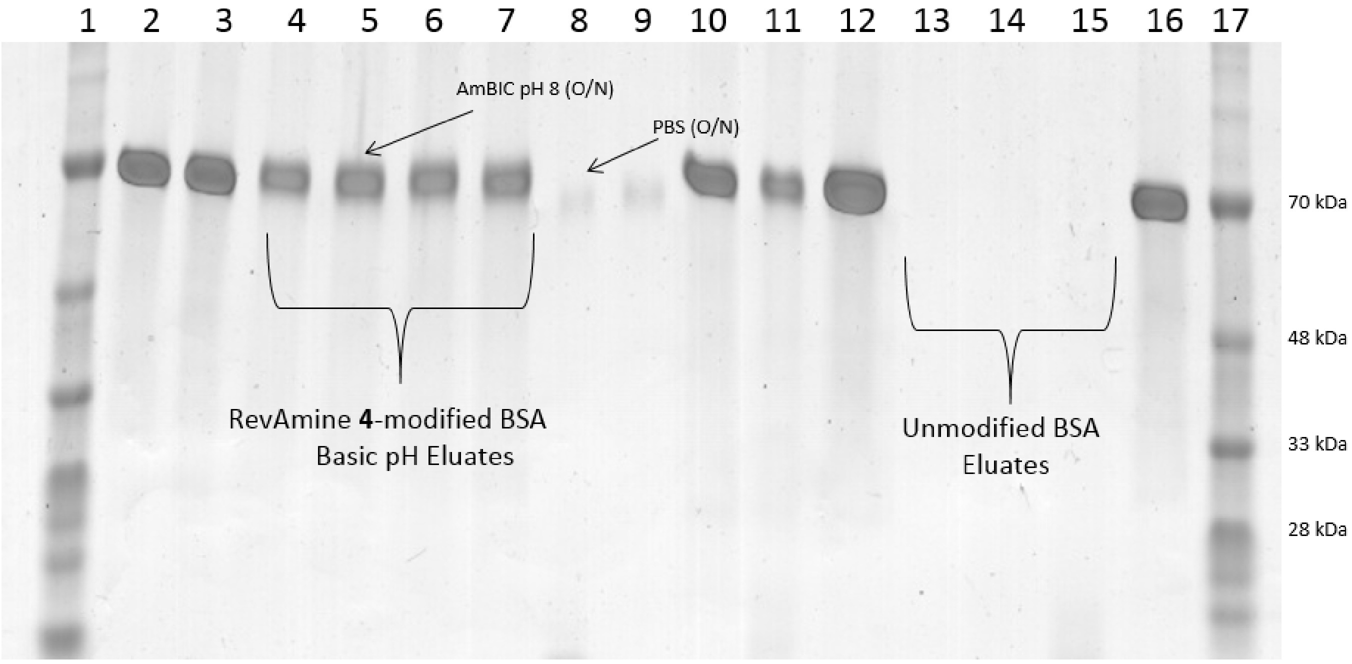

Figure S9. PAGE analysis of eluates from neutravidin capture-release of RevAmine **4**-labelled BSA. Content of the lanes are as follows: 1 & 17 = MW marker. 2, 10 & 16 = BSA. 3 = RevAmine **4**-labelled BSA. 4–8 = Eluates from neutravidin capture of RevAmine **4**-modified BSA treated as follows: 0.1% $NH_4OH_{(aq)}$ for 1 hour at RT, overnight incubation with 100mM ammonium bicarbonate at pH 8.0, 8.5 and 9.0 and with PBS pH 7.4 respectively. 9 = Beads used for lane 7 post-elution, boiled in sample loading buffer. 11 = Lane 8 (PBS) eluted beads boiled in sample loading buffer. 12 = Residual BSA in the supernatant of 13 following incubation with neutravidin beads. 13-15 = Eluates following unmodified BSA incubation with neutravidin, washing and then treating the beads overnight with PBS, pH 7.4, 100mM ammonium bicarbonate pH 9.0 or boiling in loading buffer respectively. Unless stated, all elutions were performed by incubating the beads at 4 °C.

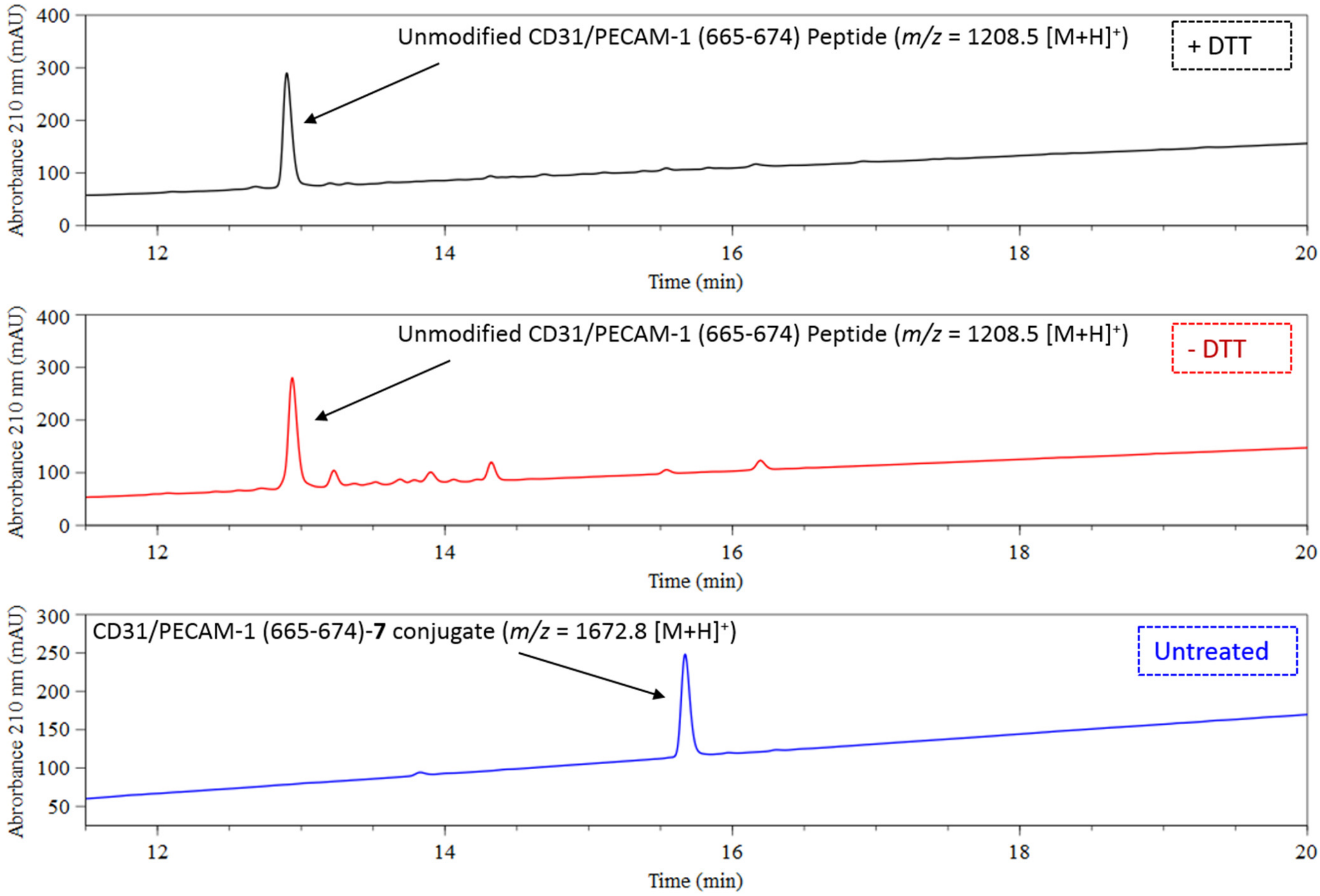

Figure S10. Improved product release in the presence of DTT. RP-HPLC analysis of CD31 (665-674)-**7** conjugate, following overnight treatment with HPLC buffer A (0.1 % formic acid (aq), blue) and 100 mM ammonium bicarbonate, pH 8, both with (black) and without (red) the presence of (5 mM) DTT.

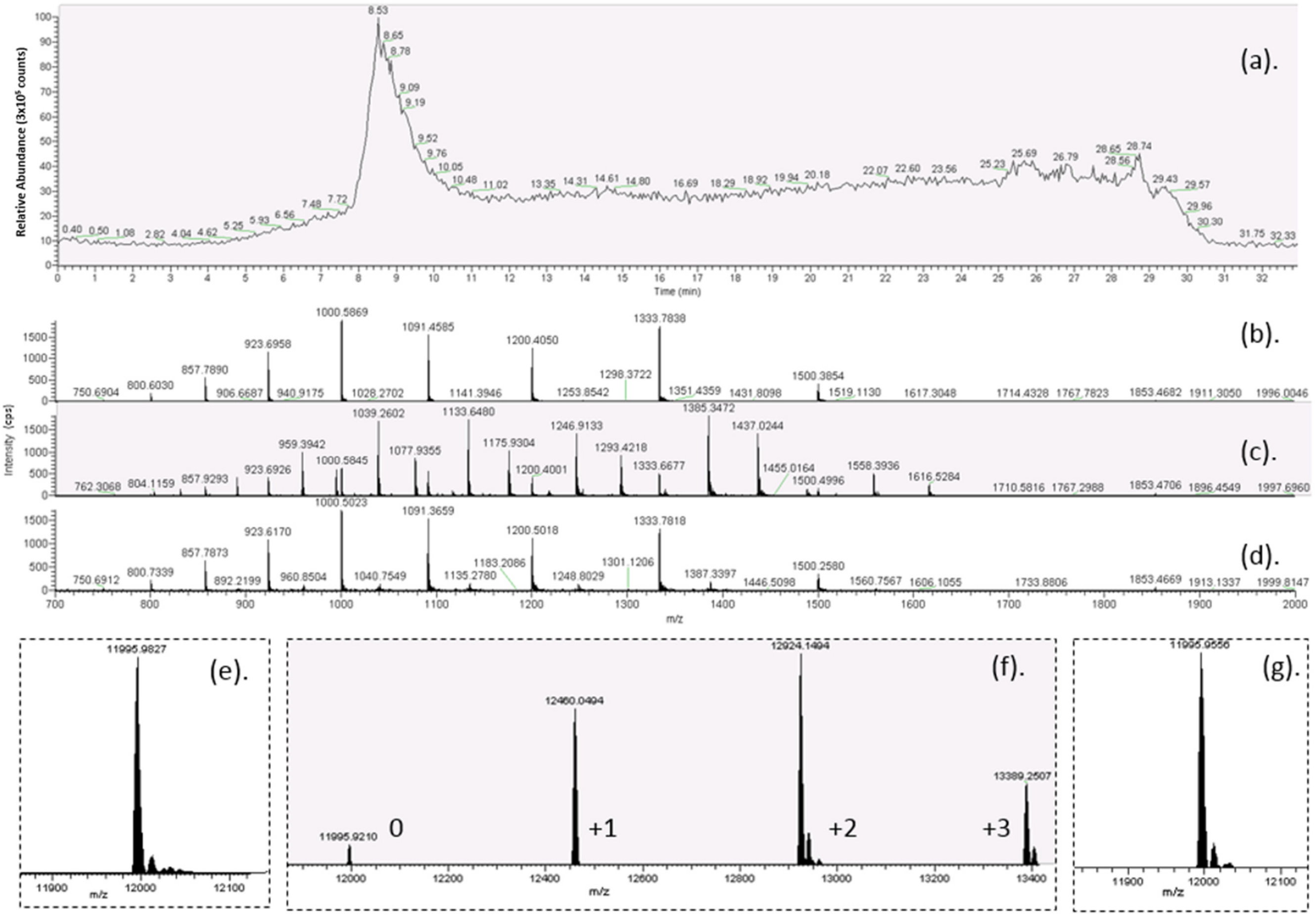

Figure S11. Trapping LC-MS data illustrating neutravidin capture and release of **7**-modified nuclease B.
(a). Total ion chromatogram for nuclease B elution with elution peak at 8.5 minutes.
Electrospray MS charge state distributions for (b) unmodified, (c) **7**-modified and (d) eluted nuclease B.
(e). Deconvoluted mass of 11995.98 for unmodified nuclease B (molecular weight (calc.) = 11995.32 Da).
(f). Range of deconvoluted masses obtained for **7**-modified nuclease B. The mass peaks are about 465 Da apart, corresponding to the mass of one **7** reversible biotin. In the case of nuclease B up to three biotins were added under the coupling conditions.
(g). Deconvoluted mass for unmodified nuclease B released from neutravidin following treatment with 200 mM ammonium bicarbonate, 20 mM DTT, 24h. The mass of 11995.96 Da is in excellent agreement with both the measured (e) and predicted unmodified mass.

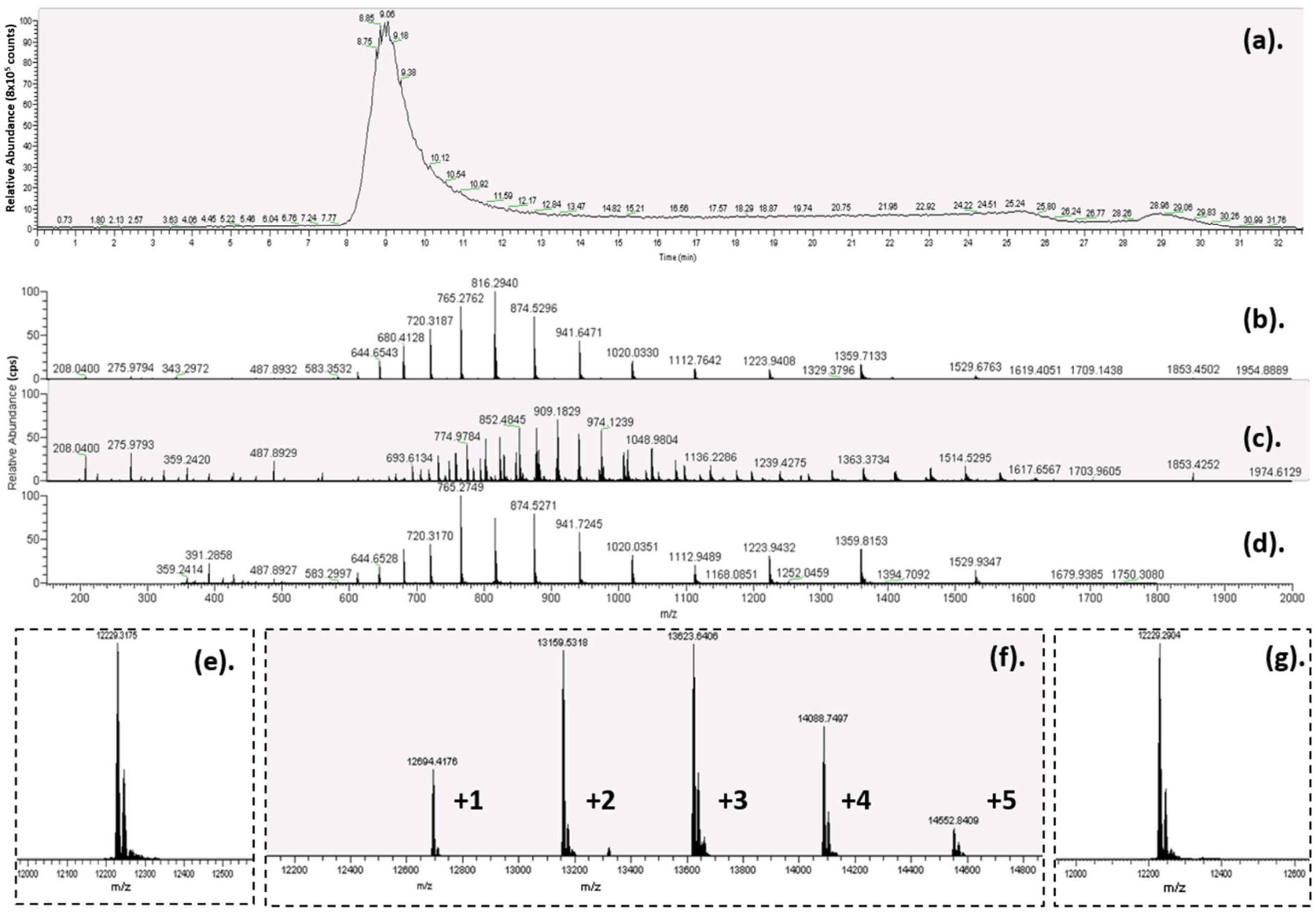

Figure S12. Trapping LC-MS data illustrating neutravidin capture and release of **7**-modified cytochrome C.
(a). Total ion chromatogram for cytochrome C elution with elution peak at 9.1 minutes.
Electrospray MS charge state distributions for (b) unmodified, (c) **7**-modified and (d) eluted cytochrome C.
(e). Deconvoluted mass of 12229.32 for unmodified nuclease B (reported molecular weight (PDB code 2B4Z) = 12230 Da)[1].
(f). Range of deconvoluted masses obtained for **7** modified cytochrome C. The mass peaks are about 465 Da apart, corresponding to the mass of one **7** reversible biotin. In the case of cytochrome C, up to five biotins were added under the coupling conditions.
(g). Deconvoluted mass for unmodified cytochrome C released from neutravidin following treatment with 200 mM ammonium bicarbonate, 20 mM DTT, 24h. The mass of 12229.29 Da is in excellent agreement with both the measured (e) and reported unmodified mass[1].

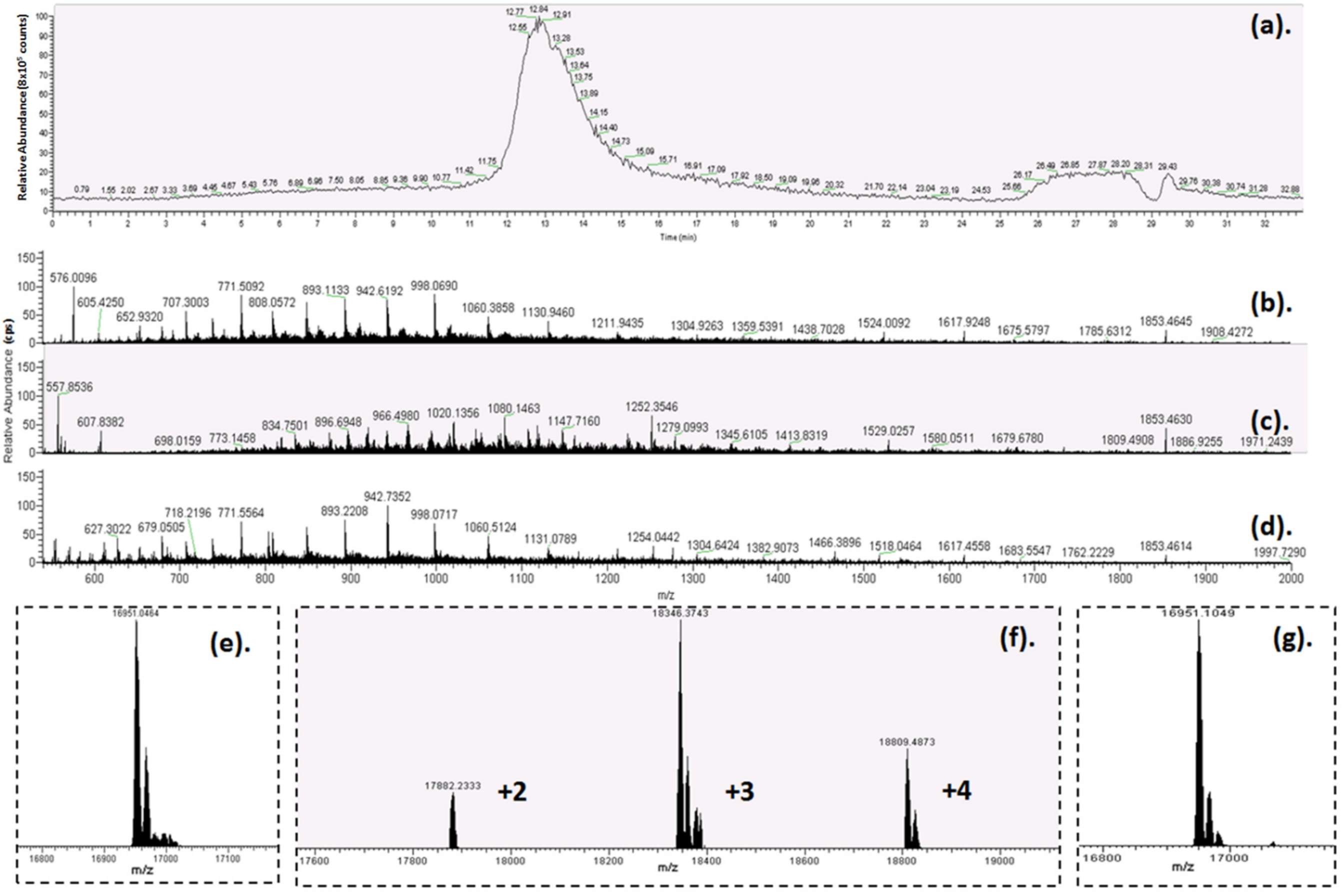

Figure S13. Trapping LC-MS data illustrating neutravidin capture and release of **7**-modified apomyoglobin.
(a). Total ion chromatogram for apomyoglobin elution with elution peak at 12.8 minutes.
Electrospray MS charge state distributions for (b) unmodified, (c) **7**-modified and (d) eluted apomyoglobin.
(e). Deconvoluted mass of 16951.05 for unmodified apomyoglobin (molecular weight (calc.) = 16951.48 Da).
(f). Range of deconvoluted masses obtained for **7** modified apomyoglobin. The mass peaks are about 465 Da apart, corresponding to the mass of one **7** reversible biotin. In the case of apomyoglobin up to four biotins were added under the coupling conditions.
(g). Deconvoluted mass for unmodified apomyoglobin released from neutravidin following treatment with 200 mM ammonium bicarbonate, 20 mM DTT, 24h. The mass of 16951.10 Da is in excellent agreement with the measured (e) and predicted unmodified mass.

Figure S14. Screenshot of the PANTHER overrepresentation test results. The gene list from table ST4 was uploaded and searched for overrepresentation (http://pantherdb.org/webservices/go/overrep.jsp) against a reference E.coli gene list targeting the cellular component annotation data set.

Analysis Summary: Please report in publication

Analysis Type: PANTHER Overrepresentation Test (release 20150430)

Annotation Version and Release Date: GO Ontology database Released 2015-08-06

Analyzed List: upload_1 (Escherichia coli) Change

Reference List: Escherichia coli (all genes in database) Change

Annotation Data Set: GO cellular component complete

☑ Use the Bonferroni correction for multiple testing

Results

	Reference list	upload_1
Mapped IDs:	4260	63
Unmapped IDs:	0	1

Export results

Displaying only results with P<0.05; click here to display all results

	Escherichia coli (REF)	upload 1				
GO cellular component complete	#	#	expected	Fold Enrichment	+/-	P value
outer membrane-bounded periplasmic space (GO:0030288)	132	11	1.95	> 5	+	4.07E-04
periplasmic space (GO:0042597)	209	12	3.09	3.88	+	5.77E-03
external encapsulating structure part (GO:0044462)	252	12	3.73	3.22	+	3.34E-02
cell envelope (GO:0030313)	255	12	3.77	3.18	+	3.72E-02
envelope (GO:0031975)	256	12	3.79	3.17	+	3.85E-02
cytosol (GO:0005829)	936	28	13.84	2.02	+	6.89E-03
cytoplasmic part (GO:0044444)	955	28	14.12	1.98	+	1.00E-02
cell part (GO:0044464)	2748	55	40.64	1.35	+	5.08E-03
cell (GO:0005623)	2752	55	40.70	1.35	+	5.40E-03
cellular_component (GO:0005575)	2816	55	41.65	1.32	+	1.38E-02
Unclassified	1444	8	21.35	.37	-	0.00E00

About | Release Information | Contact Us | System Requirements | Privacy Policy | Disclaimer

© Copyright 2016 Paul Thomas All Rights Reserved.

Table ST1. Table listing accession codes and names for the proteins identified from proteomic analysis of the neutravidin-enriched RevAmine **7** surface–labelled Ami ABC E.coli cells. Accession codes were obtained by searching the LC/MS/MS data against an in-house E.coli database using the Mascot MS/MS search tool as described below. OS = originating species.

Accession	Protein Name
E2QHT9_ECOLX	D-ribose transporter subunit RbsB OS=*Escherichia coli*
E2QGI0_ECOLX	Trigger factor OS=*Escherichia coli*
C3SFP2_ECOLX	50S ribosomal protein L9 OS=*Escherichia coli*
C3SIB2_ECOLX	50S ribosomal protein L7/L12 OS=*Escherichia coli*
C3TRK2_ECOLX	Chaperone protein DnaK OS=*Escherichia coli*
C3T122_ECOLX	Glucose-specific PTS system IIA component OS=*Escherichia coli*
C3TCN2_ECOLX	DNA-binding protein OS=*Escherichia coli*
E2QDR0_ECOLX	Phosphoglycerate kinase OS=*Escherichia coli*
C3TBH7_ECOLX	Probable thiol peroxidase OS=*Escherichia coli*
C3SQS2_ECOLX	30S ribosomal protein S7 OS=*Escherichia coli*
C3TPM7_ECOLX	Elongation factor Ts OS=*Escherichia coli*
C3SIB7_ECOLX	50S ribosomal protein L10 OS=*Escherichia coli*
C3SI42_ECOLX	DNA-binding protein OS=*Escherichia coli*
Q14F07_ECOLX	Thioredoxin 1 OS=*Escherichia coli*
C3SQY2_ECOLX	50S ribosomal protein L29 OS=*Escherichia coli*
C3TJX7_ECOLX	Alkyl hydroperoxide reductase OS=*Escherichia coli*
E2QP16_ECOLX	D-galactose-binding periplasmic protein OS=*Escherichia coli*
E2QJG1_ECOLX	Membrane protein OS=*Escherichia coli*
C3TPI7_ECOLX	Chaperone protein skp OS=*Escherichia coli*
Q548M1_ECOLX	60 kDa chaperonin OS=*Escherichia coli*
C3SX72_ECOLX	Enolase OS=*Escherichia coli*
E2QKH7_ECOLX	3-oxoacyl-[acyl-carrier-protein] reductase OS=*Escherichia coli*
C3TPL7_ECOLX	Ribosome-recycling factor OS=*Escherichia coli*
C3TRH7_ECOLX	30S ribosomal protein S20 OS=*Escherichia coli*
C3SR72_ECOLX	50S ribosomal protein L17 OS=*Escherichia coli*
C3TJN7_ECOLX	Cold shock protein OS=*Escherichia coli*
Q7BGE6_ECOLX	10 kDa chaperonin OS=*Escherichia coli*
E2QFK2_ECOLX	Peptidyl-prolyl cis-trans isomerase OS=*Escherichia coli*
E2QG20_ECOLX	Inhibitor of vertebrate lysozyme OS=*Escherichia coli*
C3SR12_ECOLX	30S ribosomal protein S8 OS=*Escherichia coli*
C3SRL7_ECOLX	Acetyl-CoA carboxylase OS=*Escherichia coli*
C3TLR7_ECOLX	DNA-binding protein HU-1 OS=*Escherichia coli*
C3SIC7_ECOLX	50S ribosomal protein L11 OS=*Escherichia coli*
C3TGB2_ECOLX	30S ribosomal protein S1 OS=*Escherichia coli*
E2QF90_ECOLX	Protease do OS=*Escherichia coli*
C3SM87_ECOLX	DNA-directed RNA polymerase subunit omega OS=*Escherichia coli*
C3SR67_ECOLX	DNA-directed RNA polymerase subunit alpha OS=*Escherichia coli*
C3SR27_ECOLX	30S ribosomal protein S5 OS=*Escherichia coli*
C3STZ7_ECOLX	30S ribosomal protein S21 OS=*Escherichia coli*
E2QL18_ECOLX	Inorganic pyrophosphatase OS=*Escherichia coli*
E2QQ39_ECOLX	Autonomous glycyl radical cofactor OS=*Escherichia coli*
C3SIC2_ECOLX	50S ribosomal protein L1 OS=*Escherichia coli*

C3T8I7_ECOLX	Glutaredoxin OS=*Escherichia coli*
E2QFJ4_ECOLX	Elongation factor Tu OS=*Escherichia coli*
C3SE75_ECOLX	Hyperosmotically inducible periplasmic protein OS=*Escherichia coli*
C3TLB7_ECOLX	Nucleoid-associated protein YbaB OS=*Escherichia coli*
C3SQT7_ECOLX	30S ribosomal protein S10 OS=*Escherichia coli*
C3SFQ7_ECOLX	30S ribosomal protein S6 OS=*Escherichia coli*
C3SQN2_ECOLX	Peptidyl-prolyl cis-trans isomerase OS=*Escherichia coli*
C3T6W2_ECOLX	Glyceraldehyde-3-phosphate dehydrogenase OS=*Escherichia coli*
E2QH68_ECOLX	Adenylate kinase OS=*Escherichia coli*
E2QL34_ECOLX	Endoribonuclease L-PSP OS=*Escherichia coli*
C3SSN7_ECOLX	Transcription elongation factor NusA OS=*Escherichia coli*
E2QFG6_ECOLX	50S ribosomal protein L24 OS=*Escherichia coli*
E2QDQ6_ECOLX	Uncharacterized protein yggE OS=*Escherichia coli*
C3TDX7_ECOLX	Acyl carrier protein OS=*Escherichia coli*
C3TPZ2_ECOLX	RNA polymerase-binding transcription factor DksA OS=*Escherichia coli*
C3SIV2_ECOLX	50S ribosomal protein L31 OS=*Escherichia coli*
C3T2X7_ECOLX	Glycerophosphodiester phosphodiesterase OS=*Escherichia coli*
E2QF45_ECOLX	Acetyltransferase component of pyruvate dehydrogenase complex OS=*Escherichia coli*
C3T887_ECOLX	Major outer membrane lipoprotein OS=*Escherichia coli*
E2QP51_ECOLX	50S ribosomal protein L25 OS=*Escherichia coli*
E2QL02_ECOLX	Peptidyl-prolyl cis-trans isomerase OS=*Escherichia coli*
E2QP76_ECOLX	Ecotin OS=*Escherichia coli*
C3T6A2_ECOLX	Cold shock protein OS=*Escherichia coli*
C3SIY7_ECOLX	Regulator of ribonuclease activity A OS=*Escherichia coli*
E2QHT6_ECOLX	D-ribose pyranase OS=*Escherichia coli*
C3STK2_ECOLX	Putative membrane protein YqjD OS=*Escherichia coli*
E2QGH0_ECOLX	UPF0234 protein YajQ OS=*Escherichia coli*
C3SQU2_ECOLX	50S ribosomal protein L3 OS=*Escherichia coli*
E2QE61_ECOLX	Outer membrane channel protein OS=*Escherichia coli*
C3TR42_ECOLX	Chaperone SurA OS=*Escherichia coli*
C3SR32_ECOLX	50S ribosomal protein L30 OS=*Escherichia coli*
C3TPT7_ECOLX	Iron-sulfur cluster insertion protein ErpA OS=*Escherichia coli*
C3SR02_ECOLX	50S ribosomal protein L5 OS=*Escherichia coli*
C3SYM7_ECOLX	Protein GrpE OS=*Escherichia coli*
C3TJ02_ECOLX	Flavodoxin OS=*Escherichia coli*
C3SMN2_ECOLX	Glutaredoxin OS=*Escherichia coli*
C3SR17_ECOLX	50S ribosomal protein L6 OS=*Escherichia coli*
E2QM03_ECOLX	Uncharacterized protein yncE OS=*Escherichia coli*
C3SME7_ECOLX	50S ribosomal protein L33 OS=*Escherichia coli*
E2QE97_ECOLX	Transaldolase OS=*Escherichia coli*
C3TPN2_ECOLX	30S ribosomal protein S2 OS=*Escherichia coli*
C3T8H7_ECOLX	Superoxide dismutase OS=*Escherichia coli*
C3SR07_ECOLX	30S ribosomal protein S14 OS=*Escherichia coli*
C3TID7_ECOLX	2,3-bisphosphoglycerate-dependent phosphoglycerate mutase OS=*Escherichia coli*
C3SYU7_ECOLX	Putative yhbH sigma 54 modulator OS=*Escherichia coli*
C3T8X2_ECOLX	H-NS/stpA-binding protein 2 OS=*Escherichia coli*

C3TGN2_ECOLX	Translation initiation factor IF-1 OS=*Escherichia coli*
E2QFP0_ECOLX	Shikimate kinase 1 OS=*Escherichia coli*
E2QQC2_ECOLX	DNA-binding protein OS=*Escherichia coli*
C3T582_ECOLX	Ferritin OS=*Escherichia coli*
C3SL92_ECOLX	ATP synthase subunit delta OS=*Escherichia coli*
C3SUG2_ECOLX	Protein ygiW OS=*Escherichia coli*
E2QMB6_ECOLX	Gns OS=*Escherichia coli*
E2QHS4_ECOLX	ATP synthase subunit b OS=*Escherichia coli*
C3SIZ2_ECOLX	Cell division protein ZapB OS=*Escherichia coli*
E2QPV8_ECOLX	Uncharacterized protein yfgD OS=*Escherichia coli*
E2QJ67_ECOLX	Maltose operon periplasmic protein OS=*Escherichia coli*
E2QII0_ECOLX	DNA protection during starvation protein OS=*Escherichia coli*
C3T132_ECOLX	PTS sugar transporter OS=*Escherichia coli*
C3SY22_ECOLX	Carbon storage regulator OS=*Escherichia coli*
C3SJ37_ECOLX	Triosephosphate isomerase OS=*Escherichia coli*
E2QPZ1_ECOLX	2Fe-2S ferredoxin OS=*Escherichia coli*
C3TE02_ECOLX	50S ribosomal protein L32 OS=*Escherichia coli*
E2QPY4_ECOLX	Nucleoside diphosphate kinase OS=*Escherichia coli*
C3SYP2_ECOLX	30S ribosomal protein S16 OS=*Escherichia coli*
E2QF48_ECOLX	Aconitate hydratase 2 OS=*Escherichia coli*
E2QPZ5_ECOLX	NifU-like protein OS=*Escherichia coli*
C3SRV3_ECOLX	Malate dehydrogenase OS=*Escherichia coli*
C3SGD7_ECOLX	Elongation factor P OS=*Escherichia coli*
C3TM07_ECOLX	Exodeoxyribonuclease 7 small subunit OS=*Escherichia coli*
C3TAA7_ECOLX	30S ribosomal subunit S22 OS=*Escherichia coli*
E2QLR3_ECOLX	Phage shock protein OS=*Escherichia coli*
E2QER0_ECOLX	Protease degQ OS=*Escherichia coli*
E2QJU0_ECOLX	Class B acid phosphatase OS=*Escherichia coli*
C3SR22_ECOLX	50S ribosomal protein L18 OS=*Escherichia coli*
E2QL19_ECOLX	ABC transporter periplasmic-binding protein ytfQ OS=*Escherichia coli*
Q3HSD9_ECOLX	Molecular chaperone OS=*Escherichia coli*
E2QNN4_ECOLX	UPF0265 protein YeeX OS=*Escherichia coli*
C3SSD2_ECOLX	ABC transporter substrate-binding protein OS=*Escherichia coli*
C3SSG7_ECOLX	50S ribosomal protein L27 OS=*Escherichia coli*
E2QLE1_ECOLX	Deoxyribose-phosphate aldolase OS=*Escherichia coli*
C3SN72_ECOLX	Cold shock dna-binding protein OS=*Escherichia coli*
E2QN43_ECOLX	Putative periplasmic or exported protein OS=*Escherichia coli*
Q14F23_ECOLX	Integration host factor subunit alpha OS=*Escherichia coli*
C3SIX2_ECOLX	ATP-dependent protease subunit HslV OS=*Escherichia coli*
C3SQV2_ECOLX	50S ribosomal protein L23 OS=*Escherichia coli*
E2QKK6_ECOLX	Putrescine-binding periplasmic protein OS=*Escherichia coli*
E2QP53_ECOLX	UPF0352 protein YejL OS=*Escherichia coli*
C3SME2_ECOLX	50S ribosomal protein L28 OS=*Escherichia coli*
E2QDQ9_ECOLX	Fructose-bisphosphate aldolase OS=*Escherichia coli*
E2QL45_ECOLX	Regulator of ribonuclease activity B OS=*Escherichia coli*
E2QFR3_ECOLX	Fe/S biogenesis protein NfuA OS=*Escherichia coli*

C3SQW2_ECOLX	30S ribosomal protein S19 OS=*Escherichia coli*
E2QLK2_ECOLX	Peptide ABC transporter substrate-binding protein OS=*Escherichia coli*
C3SZQ2_ECOLX	Iron-binding protein IscA OS=*Escherichia coli*
C3TG87_ECOLX	UPF0434 protein YcaR OS=*Escherichia coli*
C3T0B7_ECOLX	Bacterioferritin comigratory protein OS=*Escherichia coli*
E2QIT6_ECOLX	Superoxide dismutase OS=*Escherichia coli*
E2QEB5_ECOLX	RNA polymerase sigma factor RpoD OS=*Escherichia coli*
E2QFJ1_ECOLX	Bacterioferritin OS=*Escherichia coli*
E2QEL4_ECOLX	Transcription elongation factor GreA OS=*Escherichia coli*
C3T550_ECOLX	Uncharacterized protein OS=*Escherichia coli*
C3SZD2_ECOLX	Nitrogen regulatory protein P-II OS=*Escherichia coli*
C3SR47_ECOLX	50S ribosomal protein L36 OS=*Escherichia coli*
C3SSP7_ECOLX	Ribosome-binding factor A OS=*Escherichia coli*
C3SVM2_ECOLX	Ribose-5-phosphate isomerase A OS=*Escherichia coli*
E2QI48_ECOLX	Thiol:disulfide interchange protein OS=*Escherichia coli*
C3SRA2_ECOLX	Peptide deformylase OS=*Escherichia coli*
C3TQ27_ECOLX	Aspartate 1-decarboxylase OS=*Escherichia coli*
C3SQR7_ECOLX	30S ribosomal protein S12 OS=*Escherichia coli*
E2QQZ3_ECOLX	Protein-disulfide isomerase OS=*Escherichia coli*
C3TQK2_ECOLX	Cell division protein FtsZ OS=*Escherichia coli*
E2QQE1_ECOLX	S-ribosylhomocysteine lyase OS=*Escherichia coli*
Q2LD76_ECOLX	Uncharacterized protein OS=*Escherichia coli*
C3SRY2_ECOLX	30S ribosomal protein S9 OS=*Escherichia coli*
C3T2J7_ECOLX	ElaB protein OS=*Escherichia coli*
E2QID3_ECOLX	6-phosphogluconolactonase OS=*Escherichia coli*
E2QKG8_ECOLX	Ribonuclease E OS=*Escherichia coli*
E2QLE4_ECOLX	Purine nucleoside phosphorylase DeoD-type OS=*Escherichia coli*
E2QQF2_ECOLX	Glucitol/sorbitol-specific phosphotransferase enzyme OS=*Escherichia coli*
C3TF92_ECOLX	Cold shock protein CspG OS=*Escherichia coli*
C3SVN7_ECOLX	Cell division protein ZapA OS=*Escherichia coli*
C3SRI7_ECOLX	DNA-binding protein Fis OS=*Escherichia coli*
E2QFQ1_ECOLX	33 kDa chaperonin OS=*Escherichia coli*
Q2LD73_ECOLX	Cell division topological specificity factor OS=*Escherichia coli*
C3SKY2_ECOLX	Protein yifE OS=*Escherichia coli*
E2QFN8_ECOLX	Protein damX OS=*Escherichia coli*
C3TJK7_ECOLX	UPF0250 protein YbeD OS=*Escherichia coli*
E2QPV2_ECOLX	Outer membrane protein assembly factor BamC OS=*Escherichia coli*
E2QKI7_ECOLX	HIT-like protein ycfF OS=*Escherichia coli*
E2QJB4_ECOLX	Formate acetyltransferase 1 OS=*Escherichia coli*
C3TPS2_ECOLX	5'-methylthioadenosine/S-adenosylhomocysteine nucleosidase OS=*Escherichia coli*
C3TMF7_ECOLX	UPF0345 protein YaiE OS=*Escherichia coli*
C3TQA2_ECOLX	Dihydrolipoyl dehydrogenase OS=*Escherichia coli*
E2QF44_ECOLX	Pyruvate dehydrogenase E1 component OS=*Escherichia coli*
C3TE07_ECOLX	Uncharacterized protein OS=*Escherichia coli*
E2QIS4_ECOLX	Formate dehydrogenase OS=*Escherichia coli*
E2QF50_ECOLX	S-adenosylmethionine decarboxylase proenzyme OS=*Escherichia coli*

E2QJ08_ECOLX	Transcription termination/antitermination protein NusG OS=*Escherichia coli*
C3TIL2_ECOLX	Succinyl-CoA ligase [ADP-forming] subunit beta OS=*Escherichia coli*
E2QLJ9_ECOLX	Acetaldehyde dehydrogenase OS=*Escherichia coli*
E2QNC4_ECOLX	Uncharacterized lipoprotein yedD OS=*Escherichia coli*
C3SR52_ECOLX	30S ribosomal protein S13 OS=*Escherichia coli*
C3SSK2_ECOLX	ATP-dependent zinc metalloprotease FtsH OS=*Escherichia coli*
E2QE74_ECOLX	Uncharacterized protein OS=*Escherichia coli*
E2QMS8_ECOLX	Translation initiation factor IF-3 OS=*Escherichia coli*
C3T982_ECOLX	Protein ydgH OS=*Escherichia coli*
C3SQY7_ECOLX	30S ribosomal protein S17 OS=*Escherichia coli*
E2QJI7_ECOLX	*Escherichia coli* IMT2125 genomic chromosome, IMT2125 OS=*Escherichia coli*
C3T5P7_ECOLX	Probable transcriptional regulatory protein YebC OS=*Escherichia coli*
C3SR37_ECOLX	50S ribosomal protein L15 OS=*Escherichia coli*
C0KWF5_ECOLX	L-asparaginase 2 OS=*Escherichia coli*
E2QHX6_ECOLX	Uroporphyrinogen-III C-methyltransferase OS=*Escherichia coli*
B9VUA5_ECOLX	Acriflavine resistance protein A OS=*Escherichia coli*
C3SGW2_ECOLX	Protein phnA OS=*Escherichia coli*
C3SQN7_ECOLX	Protein SlyX OS=*Escherichia coli*
C3SY57_ECOLX	Regulator of plasmid mcrB operon OS=*Escherichia coli*
E2QIW0_ECOLX	Cell division protein ftsN OS=*Escherichia coli*
E2QKY3_ECOLX	Ribosome-binding ATPase YchF OS=*Escherichia coli*
E2QNI5_ECOLX	Probable transcriptional regulatory protein YeeN OS=*Escherichia coli*
E2QNP4_ECOLX	ATP phosphoribosyltransferase OS=*Escherichia coli*
E2QQ11_ECOLX	Serine hydroxymethyltransferase OS=*Escherichia coli*
C3SL97_ECOLX	ATP synthase subunit alpha OS=*Escherichia coli*
E2QK86_ECOLX	Entericidin B OS=*Escherichia coli*
E2QNB6_ECOLX	Cystine transporter subunit OS=*Escherichia coli*
C3THM2_ECOLX	Glutamine ABC transporter substrate-bindnig protein OS=*Escherichia coli*
C3TKM2_ECOLX	Peptidyl-prolyl cis-trans isomerase OS=*Escherichia coli*
E2QI20_ECOLX	Uridine phosphorylase OS=*Escherichia coli*
C3SLR2_ECOLX	Small heat shock protein IbpA OS=*Escherichia coli*
C3SD38_ECOLX	Imidazole glycerol phosphate synthase subunit HisF OS=*Escherichia coli*
C3SLB2_ECOLX	ATP synthase epsilon chain OS=*Escherichia coli*
C3SS42_ECOLX	Aerobic respiration control sensor protein ArcB OS=*Escherichia coli*
C3TRF7_ECOLX	Peptidyl-prolyl cis-trans isomerase OS=*Escherichia coli*
E2QFC5_ECOLX	Lipoprotein OS=*Escherichia coli*
E2QHK8_ECOLX	Amino acid transporter OS=*Escherichia coli*
E2QHQ2_ECOLX	Uncharacterized protein yieF OS=*Escherichia coli*
E2QI32_ECOLX	Sec-independent protein translocase protein TatB OS=*Escherichia coli*
E2QI74_ECOLX	Negative modulator of initiation of replication OS=*Escherichia coli*
E2QIU1_ECOLX	Periplasmic protein OS=*Escherichia coli*
E2QMU7_ECOLX	Osmotically-inducible lipoprotein E OS=*Escherichia coli*
E2QN35_ECOLX	ProP effector OS=*Escherichia coli*
E2QLA4_ECOLX	Putative uncharacterized protein OS=*Escherichia coli*
C3TIN2_ECOLX	Succinate dehydrogenase OS=*Escherichia coli*
C3TJK2_ECOLX	D-alanyl-D-alanine carboxypeptidase OS=*Escherichia coli*

C3TPQ2_ECOLX	UPF0325 protein YaeH OS=*Escherichia coli*
E2QDV1_ECOLX	Probable Fe(2+)-trafficking protein OS=*Escherichia coli*
E2QFB7_ECOLX	UPF0253 protein yaeP OS=*Escherichia coli*
E2QGA9_ECOLX	FrmR: Negative transcriptional regulator of formaldehyde detoxification operon OS=*Escherichia coli*
E2QHG9_ECOLX	Alkyl hydroperoxide reductase F52a subunit OS=*Escherichia coli*
E2QJ64_ECOLX	Maltose-binding periplasmic protein OS=*Escherichia coli*
E2QJQ9_ECOLX	Ferrous iron transport periplasmic protein EfeO, contains peptidase-M75 domain and (Frequently) cupredoxin-like domain OS=*Escherichia coli*
E2QLM0_ECOLX	Tryptophan synthase alpha chain OS=*Escherichia coli*
E2QNW4_ECOLX	D-tagatose-1,6-bisphosphate aldolase subunit GatY OS=*Escherichia coli*
E2QPH0_ECOLX	Histidine-binding periplasmic protein OS=*Escherichia coli*
E2QPX9_ECOLX	Membrane protein OS=*Escherichia coli*
Q933I0_ECOLX	Protein yciF OS=*Escherichia coli*

Table ST2. Table listing accession codes and names for the proteins identified from proteomic analysis of post-lysis RevAmine **7** labelled Ami ABC E.coli control cells. Accession codes were obtained by searching the LC/MS/MS data against an in-house E.coli database using the Mascot MS/MS search tool as described below. OS = originating species.

Accession	Protein Name
E2QHT9_ECOLX	D-ribose transporter subunit RbsB OS=*Escherichia coli*
E2QGI0_ECOLX	Trigger factor OS=*Escherichia coli*
C3SFP2_ECOLX	50S ribosomal protein L9 OS=*Escherichia coli*
C3SIB2_ECOLX	50S ribosomal protein L7/L12 OS=*Escherichia coli*
C3TRK2_ECOLX	Chaperone protein DnaK OS=*Escherichia coli*
C3T122_ECOLX	Glucose-specific PTS system IIA component OS=*Escherichia coli*
C3TCN2_ECOLX	DNA-binding protein OS=*Escherichia coli*
E2QDR0_ECOLX	Phosphoglycerate kinase OS=*Escherichia coli*
C3TBH7_ECOLX	Probable thiol peroxidase OS=*Escherichia coli*
C3SQS2_ECOLX	30S ribosomal protein S7 OS=*Escherichia coli*
C3TPM7_ECOLX	Elongation factor Ts OS=*Escherichia coli*
C3SIB7_ECOLX	50S ribosomal protein L10 OS=*Escherichia coli*
C3SI42_ECOLX	DNA-binding protein OS=*Escherichia coli*
Q14F07_ECOLX	Thioredoxin 1 OS=*Escherichia coli*
C3SQY2_ECOLX	50S ribosomal protein L29 OS=*Escherichia coli*
C3TJX7_ECOLX	Alkyl hydroperoxide reductase OS=*Escherichia coli*
E2QP16_ECOLX	D-galactose-binding periplasmic protein OS=*Escherichia coli*
E2QJG1_ECOLX	Membrane protein OS=*Escherichia coli*
C3TPI7_ECOLX	Chaperone protein skp OS=*Escherichia coli*
Q548M1_ECOLX	60 kDa chaperonin OS=*Escherichia coli*
C3SX72_ECOLX	Enolase OS=*Escherichia coli*
E2QKH7_ECOLX	3-oxoacyl-[acyl-carrier-protein] reductase OS=*Escherichia coli*
C3TPL7_ECOLX	Ribosome-recycling factor OS=*Escherichia coli*
C3TRH7_ECOLX	30S ribosomal protein S20 OS=*Escherichia coli*
C3SR72_ECOLX	50S ribosomal protein L17 OS=*Escherichia coli*
C3TJN7_ECOLX	Cold shock protein OS=*Escherichia coli*
Q7BGE6_ECOLX	10 kDa chaperonin OS=*Escherichia coli*
E2QFK2_ECOLX	Peptidyl-prolyl cis-trans isomerase OS=*Escherichia coli*
E2QG20_ECOLX	Inhibitor of vertebrate lysozyme OS=*Escherichia coli*
C3SR12_ECOLX	30S ribosomal protein S8 OS=*Escherichia coli*
C3SRL7_ECOLX	Acetyl-CoA carboxylase OS=*Escherichia coli*
C3TLR7_ECOLX	DNA-binding protein HU-1 OS=*Escherichia coli*
C3SIC7_ECOLX	50S ribosomal protein L11 OS=*Escherichia coli*
C3TGB2_ECOLX	30S ribosomal protein S1 OS=*Escherichia coli*
E2QF90_ECOLX	Protease do OS=*Escherichia coli*
C3SM87_ECOLX	DNA-directed RNA polymerase subunit omega OS=*Escherichia coli*
C3SR67_ECOLX	DNA-directed RNA polymerase subunit alpha OS=*Escherichia coli*
C3SR27_ECOLX	30S ribosomal protein S5 OS=*Escherichia coli*
C3STZ7_ECOLX	30S ribosomal protein S21 OS=*Escherichia coli*
E2QL18_ECOLX	Inorganic pyrophosphatase OS=*Escherichia coli*
E2QQ39_ECOLX	Autonomous glycyl radical cofactor OS=*Escherichia coli*

C3SIC2_ECOLX	50S ribosomal protein L1 OS=*Escherichia coli*
C3T8I7_ECOLX	Glutaredoxin OS=*Escherichia coli*
E2QFJ4_ECOLX	Elongation factor Tu OS=*Escherichia coli*
C3SE75_ECOLX	Hyperosmotically inducible periplasmic protein OS=*Escherichia coli*
C3TLB7_ECOLX	Nucleoid-associated protein YbaB OS=*Escherichia coli*
C3SQT7_ECOLX	30S ribosomal protein S10 OS=*Escherichia coli*
C3SFQ7_ECOLX	30S ribosomal protein S6 OS=*Escherichia coli*
C3SQN2_ECOLX	Peptidyl-prolyl cis-trans isomerase OS=*Escherichia coli*
C3T6W2_ECOLX	Glyceraldehyde-3-phosphate dehydrogenase OS=*Escherichia coli*
E2QH68_ECOLX	Adenylate kinase OS=*Escherichia coli*
C3SQU7_ECOLX	50S ribosomal protein L4 OS=*Escherichia coli*
E2QL34_ECOLX	Endoribonuclease L-PSP OS=*Escherichia coli*
C3SSN7_ECOLX	Transcription elongation factor NusA OS=*Escherichia coli*
E2QFG6_ECOLX	50S ribosomal protein L24 OS=*Escherichia coli*
C3TDX7_ECOLX	Acyl carrier protein OS=*Escherichia coli*
C3TPZ2_ECOLX	RNA polymerase-binding transcription factor DksA OS=*Escherichia coli*
C3SIV2_ECOLX	50S ribosomal protein L31 OS=*Escherichia coli*
C3T2X7_ECOLX	Glycerophosphodiester phosphodiesterase OS=*Escherichia coli*
E2QF45_ECOLX	Acetyltransferase component of pyruvate dehydrogenase complex OS=*Escherichia coli*
C3T887_ECOLX	Major outer membrane lipoprotein OS=*Escherichia coli*
E2QP51_ECOLX	50S ribosomal protein L25 OS=*Escherichia coli*
E2QL02_ECOLX	Peptidyl-prolyl cis-trans isomerase OS=*Escherichia coli*
E2QP76_ECOLX	Ecotin OS=*Escherichia coli*
C3T6A2_ECOLX	Cold shock protein OS=*Escherichia coli*
C3SIY7_ECOLX	Regulator of ribonuclease activity A OS=*Escherichia coli*
E2QHT6_ECOLX	D-ribose pyranase OS=*Escherichia coli*
C3STK2_ECOLX	Putative membrane protein YqjD OS=*Escherichia coli*
E2QGH0_ECOLX	UPF0234 protein YajQ OS=*Escherichia coli*
C3SQU2_ECOLX	50S ribosomal protein L3 OS=*Escherichia coli*
E2QE61_ECOLX	Outer membrane channel protein OS=*Escherichia coli*
C3TR42_ECOLX	Chaperone SurA OS=*Escherichia coli*
C3SR32_ECOLX	50S ribosomal protein L30 OS=*Escherichia coli*
C3TPT7_ECOLX	Iron-sulfur cluster insertion protein ErpA OS=*Escherichia coli*
C3SR02_ECOLX	50S ribosomal protein L5 OS=*Escherichia coli*
C3SYM7_ECOLX	Protein GrpE OS=*Escherichia coli*
C3TJ02_ECOLX	Flavodoxin OS=*Escherichia coli*
C3SMN2_ECOLX	Glutaredoxin OS=*Escherichia coli*
C3SR17_ECOLX	50S ribosomal protein L6 OS=*Escherichia coli*
E2QM03_ECOLX	Uncharacterized protein yncE OS=*Escherichia coli*
C3SME7_ECOLX	50S ribosomal protein L33 OS=*Escherichia coli*
E2QE97_ECOLX	Transaldolase OS=*Escherichia coli*
C3TPN2_ECOLX	30S ribosomal protein S2 OS=*Escherichia coli*
C3T8H7_ECOLX	Superoxide dismutase OS=*Escherichia coli*
C3SR07_ECOLX	30S ribosomal protein S14 OS=*Escherichia coli*
C3TID7_ECOLX	2,3-bisphosphoglycerate-dependent phosphoglycerate mutase OS=*Escherichia coli*
C3SYU7_ECOLX	Putative yhbH sigma 54 modulator OS=*Escherichia coli*
C3T8X2_ECOLX	H-NS/stpA-binding protein 2 OS=*Escherichia coli*

C3TGN2_ECOLX	Translation initiation factor IF-1 OS=*Escherichia coli*
E2QFP0_ECOLX	Shikimate kinase 1 OS=*Escherichia coli*
E2QQC2_ECOLX	DNA-binding protein OS=*Escherichia coli*
C3T582_ECOLX	Ferritin OS=*Escherichia coli*
C3SL92_ECOLX	ATP synthase subunit delta OS=*Escherichia coli*
C3SUG2_ECOLX	Protein ygiW OS=*Escherichia coli*
E2QMB6_ECOLX	Gns OS=*Escherichia coli*
E2QHS4_ECOLX	ATP synthase subunit b OS=*Escherichia coli*
C3SIZ2_ECOLX	Cell division protein ZapB OS=*Escherichia coli*
E2QPV8_ECOLX	Uncharacterized protein yfgD OS=*Escherichia coli*
E2QJ67_ECOLX	Maltose operon periplasmic protein OS=*Escherichia coli*
E2QII0_ECOLX	DNA protection during starvation protein OS=*Escherichia coli*
C3T132_ECOLX	PTS sugar transporter OS=*Escherichia coli*
C3SY22_ECOLX	Carbon storage regulator OS=*Escherichia coli*
C3SJ37_ECOLX	Triosephosphate isomerase OS=*Escherichia coli*
E2QPZ1_ECOLX	2Fe-2S ferredoxin OS=*Escherichia coli*
E2QPY4_ECOLX	Nucleoside diphosphate kinase OS=*Escherichia coli*
C3TLA7_ECOLX	Chaperone protein HtpG OS=*Escherichia coli*
C3SYP2_ECOLX	30S ribosomal protein S16 OS=*Escherichia coli*
E2QF48_ECOLX	Aconitate hydratase 2 OS=*Escherichia coli*
E2QPZ5_ECOLX	NifU-like protein OS=*Escherichia coli*
C3SRV3_ECOLX	Malate dehydrogenase OS=*Escherichia coli*
C3SGD7_ECOLX	Elongation factor P OS=*Escherichia coli*
C3TM07_ECOLX	Exodeoxyribonuclease 7 small subunit OS=*Escherichia coli*
C3TAA7_ECOLX	30S ribosomal subunit S22 OS=*Escherichia coli*
E2QLR3_ECOLX	Phage shock protein OS=*Escherichia coli*
E2QPK3_ECOLX	UPF0381 protein yfcZ OS=*Escherichia coli*
C3SR22_ECOLX	50S ribosomal protein L18 OS=*Escherichia coli*
E2QL19_ECOLX	ABC transporter periplasmic-binding protein ytfQ OS=*Escherichia coli*
Q3HSD9_ECOLX	Molecular chaperone OS=*Escherichia coli*
E2QNN4_ECOLX	UPF0265 protein YeeX OS=*Escherichia coli*
C3SSD2_ECOLX	ABC transporter substrate-binding protein OS=*Escherichia coli*
C3SSG7_ECOLX	50S ribosomal protein L27 OS=*Escherichia coli*
C3SMC7_ECOLX	Deoxyuridine 5'-triphosphate nucleotidohydrolase OS=*Escherichia coli*
C3TM82_ECOLX	Preprotein translocase subunit YajC OS=*Escherichia coli*
E2QLE1_ECOLX	Deoxyribose-phosphate aldolase OS=*Escherichia coli*
C3SN72_ECOLX	Cold shock dna-binding protein OS=*Escherichia coli*
E2QN43_ECOLX	Putative periplasmic or exported protein OS=*Escherichia coli*
Q14F23_ECOLX	Integration host factor subunit alpha OS=*Escherichia coli*
C3SIX2_ECOLX	ATP-dependent protease subunit HslV OS=*Escherichia coli*
C3SQV2_ECOLX	50S ribosomal protein L23 OS=*Escherichia coli*
E2QKK6_ECOLX	Putrescine-binding periplasmic protein OS=*Escherichia coli*
E2QP53_ECOLX	UPF0352 protein YejL OS=*Escherichia coli*
C3SME2_ECOLX	50S ribosomal protein L28 OS=*Escherichia coli*
E2QDQ9_ECOLX	Fructose-bisphosphate aldolase OS=*Escherichia coli*
E2QL45_ECOLX	Regulator of ribonuclease activity B OS=*Escherichia coli*
E2QFR3_ECOLX	Fe/S biogenesis protein NfuA OS=*Escherichia coli*

C3SQX7_ECOLX	50S ribosomal protein L16 OS=*Escherichia coli*
C3SZQ2_ECOLX	Iron-binding protein IscA OS=*Escherichia coli*
C3TG87_ECOLX	UPF0434 protein YcaR OS=*Escherichia coli*
C3T0B7_ECOLX	Bacterioferritin comigratory protein OS=*Escherichia coli*
E2QIT6_ECOLX	Superoxide dismutase OS=*Escherichia coli*
E2QFJ1_ECOLX	Bacterioferritin OS=*Escherichia coli*
E2QEL4_ECOLX	Transcription elongation factor GreA OS=*Escherichia coli*
C3T550_ECOLX	Uncharacterized protein OS=*Escherichia coli*
C3SZD2_ECOLX	Nitrogen regulatory protein P-II OS=*Escherichia coli*
C3SR47_ECOLX	50S ribosomal protein L36 OS=*Escherichia coli*
C3SSP7_ECOLX	Ribosome-binding factor A OS=*Escherichia coli*
C3SVM2_ECOLX	Ribose-5-phosphate isomerase A OS=*Escherichia coli*
E2QI48_ECOLX	Thiol:disulfide interchange protein OS=*Escherichia coli*
C3SRA2_ECOLX	Peptide deformylase OS=*Escherichia coli*
C3TQ27_ECOLX	Aspartate 1-decarboxylase OS=*Escherichia coli*
C3SQR7_ECOLX	30S ribosomal protein S12 OS=*Escherichia coli*
C3TQK2_ECOLX	Cell division protein FtsZ OS=*Escherichia coli*
E2QQE1_ECOLX	S-ribosylhomocysteine lyase OS=*Escherichia coli*
C3SRY2_ECOLX	30S ribosomal protein S9 OS=*Escherichia coli*
C3T2J7_ECOLX	ElaB protein OS=*Escherichia coli*
E2QID3_ECOLX	6-phosphogluconolactonase OS=*Escherichia coli*
E2QKG8_ECOLX	Ribonuclease E OS=*Escherichia coli*
E2QLE4_ECOLX	Purine nucleoside phosphorylase DeoD-type OS=*Escherichia coli*
E2QQF2_ECOLX	Glucitol/sorbitol-specific phosphotransferase enzyme OS=*Escherichia coli*
C3SLA7_ECOLX	ATP synthase subunit beta OS=*Escherichia coli*
E2QMK5_ECOLX	Membrane protein OS=*Escherichia coli*
C3TF92_ECOLX	Cold shock protein CspG OS=*Escherichia coli*
C3SVN7_ECOLX	Cell division protein ZapA OS=*Escherichia coli*
C3SRI7_ECOLX	DNA-binding protein Fis OS=*Escherichia coli*
E2QFQ1_ECOLX	33 kDa chaperonin OS=*Escherichia coli*
Q2LD73_ECOLX	Cell division topological specificity factor OS=*Escherichia coli*
C3SKY2_ECOLX	Protein yifE OS=*Escherichia coli*
E2QFN8_ECOLX	Protein damX OS=*Escherichia coli*
C3SQS7_ECOLX	Elongation factor G OS=*Escherichia coli*
C3SZN7_ECOLX	HTH-type transcriptional regulator IscR OS=*Escherichia coli*
E2QEQ4_ECOLX	Clp protease ClpP OS=*Escherichia coli*
E2QJB4_ECOLX	Formate acetyltransferase 1 OS=*Escherichia coli*
C3TPS2_ECOLX	5'-methylthioadenosine/S-adenosylhomocysteine nucleosidase OS=*Escherichia coli*
C3TMF7_ECOLX	UPF0345 protein YaiE OS=*Escherichia coli*
C3TQA2_ECOLX	Dihydrolipoyl dehydrogenase OS=*Escherichia coli*
E2QF44_ECOLX	Pyruvate dehydrogenase E1 component OS=*Escherichia coli*
C3TE07_ECOLX	Uncharacterized protein OS=*Escherichia coli*
E2QJ08_ECOLX	Transcription termination/antitermination protein NusG OS=*Escherichia coli*
C3TIL2_ECOLX	Succinyl-CoA ligase [ADP-forming] subunit beta OS=*Escherichia coli*
E2QLJ9_ECOLX	Acetaldehyde dehydrogenase OS=*Escherichia coli*
E2QNC4_ECOLX	Uncharacterized lipoprotein yedD OS=*Escherichia coli*
C3SR52_ECOLX	30S ribosomal protein S13 OS=*Escherichia coli*

C3T137_ECOLX	Cysteine synthase OS=*Escherichia coli*
E2QQE8_ECOLX	Competence damage-inducible protein A OS=*Escherichia coli*
C3T5P7_ECOLX	Probable transcriptional regulatory protein YebC OS=*Escherichia coli*
C3SFP7_ECOLX	30S ribosomal protein S18 OS=*Escherichia coli*
E2QHD8_ECOLX	Dihydropteridine reductase OS=*Escherichia coli*
C3TM42_ECOLX	6,7-dimethyl-8-ribityllumazine synthase OS=*Escherichia coli*
C3SL97_ECOLX	ATP synthase subunit alpha OS=*Escherichia coli*
E2QK86_ECOLX	Entericidin B OS=*Escherichia coli*
C3T127_ECOLX	Phosphoenolpyruvate-protein phosphotransferase OS=*Escherichia coli*
E2QHX8_ECOLX	Porphobilinogen deaminase OS=*Escherichia coli*
C3SHF2_ECOLX	Single-stranded DNA-binding protein OS=*Escherichia coli*
C3SQA7_ECOLX	Ribulose-phosphate 3-epimerase OS=*Escherichia coli*
C3SSC7_ECOLX	Phospholipid ABC transporter substrate-binding protein OS=*Escherichia coli*
E2QFX6_ECOLX	DcrB protein OS=*Escherichia coli*
E2QH48_ECOLX	Uncharacterized lipoprotein ybaY OS=*Escherichia coli*
E2QJ37_ECOLX	Isocitrate lyase OS=*Escherichia coli*
E2QKH6_ECOLX	Malonyl CoA-acyl carrier protein transacylase OS=*Escherichia coli*
E2QQB8_ECOLX	Peptidoglycan-binding protein OS=*Escherichia coli*
E2QFC0_ECOLX	Copper homeostasis protein cutF OS=*Escherichia coli*
E2QIA0_ECOLX	Dihydrolipoyllysine-residue succinyltransferase component of 2-oxoglutarate dehydrogenase complex OS=*Escherichia coli*
E2QIB4_ECOLX	18K peptidoglycan-associated outer membrane lipoprotein OS=*Escherichia coli*
E2QNY1_ECOLX	Antiporter OS=*Escherichia coli*
E2QP37_ECOLX	Elongation factor P-like protein OS=*Escherichia coli*
Q6KCW9_ECOLX	ABC transporter permease OS=*Escherichia coli*

Table ST3. Table listing accession codes, protein names and gene names for the unique surface proteins identified by subtracting the proteins identified in the post-lysis labelling Mascot search from those identified in the surface labelling Mascot search. Gene names were generated using the DAVID Gene Accession Conversion Tool (https://david.ncifcrf.gov/content.jsp?file=conversion.html).

Accession	Protein Name	Gene Name
B9VUA5_ECOLX	Multidrug transporter	acrA
C0KWF5_ECOLX	L-asparaginase II	ansB
C3SD38_ECOLX	Imidazole glycerol phosphate synthase subunit HisF	hisF
C3SGW2_ECOLX	Uncharacterized protein	phnA
C3SLB2_ECOLX	ATP synthase epsilon chain	atpC
C3SLR2_ECOLX	Small heat shock protein IbpA	ibpA
C3SQN7_ECOLX	Protein SlyX	slyX
C3SQW2_ECOLX	30S ribosomal protein S19	rpsS
C3SQY7_ECOLX	30S ribosomal protein S17	rpsQ
C3SR37_ECOLX	50S ribosomal protein L15	rplO
C3SS42_ECOLX	Aerobic respiration control sensor protein ArcB	arcB
C3SSK2_ECOLX	ATP-dependent zinc metalloprotease FtsH	ftsH
C3SY57_ECOLX	Transcriptional repressor mprA	mprA
C3T982_ECOLX	Uncharacterized protein	ydgH
C3TE02_ECOLX	50S ribosomal protein L32	rpmF
C3THM2_ECOLX	Periplasmic glutamine-binding protein	glnH
C3TIN2_ECOLX	Succinate dehydrogenase, iron sulfur protein	sdhB
C3TJK2_ECOLX	Penicillin-binding protein 5	dacA
C3TJK7_ECOLX	UPF0250 protein YbeD	ybeD
C3TKM2_ECOLX	Peptidyl-prolyl cis-trans isomerase	ppiB
C3TPQ2_ECOLX	UPF0325 protein YaeH	yaeH
C3TRF7_ECOLX	Peptidyl-prolyl cis-trans isomerase	fkpB
E2QDQ6_ECOLX	Uncharacterized protein yggE	yggE
E2QDV1_ECOLX	Probable Fe(2+)-trafficking protein	yggX
E2QE74_ECOLX	Uncharacterized protein	yqiC
E2QEB5_ECOLX	RNA polymerase sigma factor RpoD	rpoD
E2QER0_ECOLX	Protease degQ	degQ
E2QF50_ECOLX	S-adenosylmethionine decarboxylase proenzyme	speD
E2QFB7_ECOLX	UPF0253 protein yaeP	yaeP
E2QFC5_ECOLX	Lipoprotein	metQ
E2QGA9_ECOLX	FrmR: Negative transcriptional regulator of formaldehyde detoxification operon	frmR
E2QHG9_ECOLX	Alkyl hydroperoxide reductase protein F	ahpF
E2QHK8_ECOLX	Glutamate/aspartate periplasmic-binding protein	gltI
E2QHQ2_ECOLX	Uncharacterized protein yieF	yieF
E2QHX6_ECOLX	Uroporphyrinogen-III C-methyltransferase	hemX
E2QI20_ECOLX	Uridine phosphorylase	udp
E2QI32_ECOLX	Sec-independent protein translocase protein TatB	tatB
E2QI74_ECOLX	Negative modulator of initiation of replication	seqA
E2QIS4_ECOLX	Formate dehydrogenase-O, iron-sulfur subunit	fdoH
E2QIU1_ECOLX	Periplasmic protein cpxP	cpxP

E2QIW0_ECOLX	Cell division protein ftsN	ftsN
E2QJ64_ECOLX	Maltose-binding periplasmic protein	malE
E2QJI7_ECOLX	Gns	gnsA
E2QJQ9_ECOLX	UPF0409 protein ycdO	efeO
E2QJU0_ECOLX	Class B acid phosphatase	aphA
E2QKI7_ECOLX	Purine nucleoside phosphoramidase	hinT
E2QKY3_ECOLX	Ribosome-binding ATPase YchF	ychF
E2QLA4_ECOLX	Putative uncharacterized protein	LF82_736
E2QLK2_ECOLX	Periplasmic oligopeptide-binding protein	oppA
E2QLM0_ECOLX	Tryptophan synthase alpha chain	trpA
E2QMS8_ECOLX	Translation initiation factor IF-3	infC
E2QMU7_ECOLX	Transcriptional regulator	osmE
E2QN35_ECOLX	ProP effector	proQ
E2QNB6_ECOLX	Cystine-binding periplasmic protein	fliY
E2QNI5_ECOLX	Probable transcriptional regulatory protein YeeN	yeen
E2QNP4_ECOLX	ATP phosphoribosyltransferase	hisG
E2QNW4_ECOLX	D-tagatose-1,6-bisphosphate aldolase subunit GatY	gatY
E2QPH0_ECOLX	Histidine-binding periplasmic protein	hisJ
E2QPV2_ECOLX	Outer membrane protein assembly factor BamC	bamC
E2QPX9_ECOLX	UPF0070 protein yfgM	yfgM
E2QQ11_ECOLX	Serine hydroxymethyltransferase	glyA
E2QQZ3_ECOLX	Thiol:disulfide interchange protein DsbC	dsbC
Q2LD76_ECOLX	Uncharacterized protein	ymgD
Q933I0_ECOLX	YciF protein	yciF

General experimental procedures:

Materials

HA peptide (>97% by HPLC) was obtained from Sigma. Peptide CD31 (665-674) was synthesised in-house using an ABI 431A peptide synthesiser and Fmoc-based chemistry (>97% pure by HPLC). Both peptides were desalted (Sephadex G-10) into 5% aqueous acetonitrile (MeCN) at 2mg/ml and 100 µL aliquots dried *in vacuo* (Speed Vac, Savant), then stored at -20°C until further use. All other reagents were from Sigma unless stated.

Synthetic Procedures

(4). 2-((2-((((2,5-dioxopyrrolidin-1-yl)oxy)carbonyl)oxy)ethyl)sulfonyl)ethyl 5-((3a*S*,4*S*,6a*R*)-2-oxohexahydro-1*H*-thieno[3,4-*d*]imidazol-4-yl)pentanoate

Biotin (0.391 g, 1.6 mmol), DCC (0.392 g, 1.9 mmol) and DMAP (0.028 g, 0.23 mmol) were added sequentially to a solution of 2,2'-sulfonylbis(ethan-1-ol) (0.247g, 1.6 mmol) in dry DCM (12 mL) under an atmosphere of nitrogen. The resulting suspension was stirred at room temperature for 9 days. The solvent was removed under reduced pressure and the residue was passed through a silica plug (DCM:MeOH, 9:1) to give crude 2-((2-hydroxyethyl)sulfonyl)ethyl 5-((3a*S*,4*S*,6a*R*)-2-oxohexahydro-1*H*-thieno[3,4-*d*]imidazol-4-yl)pentanoate (0.444 g).

Bis(2,5-dioxopyrrolidin-1-yl) carbonate (0.074 g, 0.281 mmol) in MeCN (1 mL) was added to a solution of 2-((2-hydroxyethyl)sulfonyl)ethyl 5-((3a*S*,4*S*,6a*R*)-2-oxohexahydro-1*H*-thieno[3,4-*d*]imidazol-4-yl)pentanoate (0.100 g, 0.263 mmol), as prepared above, and pyridine (0.08 mL, 0.263 mmol) in MeCN (2 mL) under an atmosphere of nitrogen. The reaction was stirred for 30 minutes at room temperature, the solvent removed under reduced pressure and of the resulting white solid (0.105 g), 50 mg was purified by solid phase extraction (ISOLUTE© 101, Biotage) to yield a yellow oil (16.0 mg). HRMS (ES+) $[M+H]^+$: calcd $C_{19}H_{28}N_3O_{10}S_2^+$: 522.1211; observed: 522.1208 (Figure S1 & S2).

(7). 2-((2-((((2,5-dioxopyrrolidin-1-yl)oxy)carbonyl)oxy)ethyl)sulfonyl)ethyl 2-(5-((3a*S*,4*S*,6a*R*)-2-oxohexahydro-1*H*-thieno[3,4-*d*]imidazol-4-yl)pentanoyl)hydrazine-1-carboxylate

Biotin hydrazide (26 mg, 0.1 mmol), bis(2,5-dioxopyrrolidin-1-yl) (sulfonylbis(ethane-2,1-diyl)) bis(carbonate) (44 mg, 0.1 mmol) and DMSO (200 µL) were added sequentially to a reaction vessel. After one hour at room temperature the reaction was judged complete. Analysis by HPLC showed a major peak corresponding to 2-((2-((((2,5-dioxopyrrolidin-1-yl)oxy)carbonyl)oxy)ethyl)sulfonyl)ethyl 2-(5-((3a*S*,4*S*,6a*R*)-2-oxohexahydro-1*H*-thieno[3,4-*d*]imidazol-4-yl)pentanoyl)hydrazine-1-carboxylate. The reagent was purified as per **4** to yield a yellow oil (18.0 mg). HRMS (ES+) $[M+H]^+$: calcd $C_{20}H_{30}N_5O_{11}S_2^+$: 580.1378; observed: 580.1369 (Figure S3 & S4).

RevAmine Peptide Coupling

Peptides were reconstituted at 83 mM in 100 mM sodium phosphate buffer, pH 7.4, before addition of the RevAmine reagent (**4** or **7**) as a 100 mM solution in dry DMSO in 2-fold molar excess (3:5, v:v). The reaction was performed for 2 h at RT with occasional mixing. Depending on product purity, the reaction mixes were either used directly or the modified peptides isolated by RP-HPLC before further analysis.

Analytical Procedures

Analytical RP-HPLC

To analyse reaction products and collect peptides for futher analysis by HPLC and MS, analytical reversed-phase HPLC was performed using an Agilent 1100 HPLC system fitted with a 2.1 X 150 mm ACE 3 C18 analytical column (Hichrom Ltd, UK), operating at a flow rate of 0.2 mL/min. The standard gradient conditions were 5-60% buffer B in buffer A over 27 min. Buffer A: 0.1% formic acid in water; Buffer B: 0.1% formic acid in acetonitrile (MeCN). Fractions were collected manually (typically 200 – 500 mAU @ 210 nm with 30 seconds peak width) at the expected elution times and dried *in vacuo.* Dried peptide fractions were either used directly or stored at -20ºC until required.

Base Cleavage of RevAmine-modified Peptides

Dried HPLC elution peaks were reconstituted in 50 µL of the appropriate solution, either ammonium hydroxide (aq), 1% - 0.01%; 100 mM ammonium bicarbonate, pH 8.0, 8.5 or 9.0 or phosphate buffer (100 mM, pH 7.4 as a control). All incubations were carried-out at room temperature and times varied from 10 minutes (1% NH4OH) to 18 h (pH 8.0 ammonium bicarbonate). After incubation all samples were acidified by addition of 1/10th volume 10% (v/v) aqueous trifluoroacetic acid (TFA) before a fraction (1 – 20 µL) was injected onto analytical HPLC and/or subjected to infusion MS analysis (below).

MALDI MS Analysis of Peptides

Analysis was performed by matrix-assisted laser desorption ionization-time of flight mass spectrometry (MALDI-TOF-MS) using a Voyager DE-STR (AB Sciex) operating in reflector mode (21 kV accelerating voltage, 50 ns delay time). Sample spots were prepare by mixing 1 µL of α-Cyano-4-hydroxycinnamic acid matrix (10 mg/ml in 50% (v/v) MeCN, containing 0.1% (v/v) TFA) with an equal volume of HPLC peak eluate and then spotting (2 X 1 µL) of the mixture onto the MALDI sample plate with air-drying. Positive ion peptide mass spectra were typically acquired over a m/z range of 400–4,000 with 50 laser shots per spectrum. External calibration was performed using manufacturer peptide standards (AB Sciex).

Infusion Electrospray MS Analysis

Electrospray data was acquired using an LTQ-FT mass spectrometer (Thermo) with a FT-MS resolution setting of 100,000 at m/z = 400 and an injection target value of 1,000,000. Infusion spray analyses were performed on 5-10 µL of collected HPLC fractions using medium 'nanoES' spray capillaries (Thermo) for offline nanospray mass spectrometry in positive mode at 1 kV.

Modification of Bovine Serum Albumin (BSA) with RevAmine 4

Bovine serum albumin (200 µL, 0.6 mM in phosphate buffer, pH 7.4) was modified via addition of RevAmine 4 (25 µL, 100 mM) in dry DMSO. The reaction was allowed to proceed for 1h at RT. The product was desalted into water for subsequent analysis using PD Spin Trap™ G25 (GE Healthcare, UK) as per the manufacturers instructions. The elution volume was 280 µL.

MALDI MS Analysis of BSA

Samples of desalted **4**-biotinylated and unbiotinylated control BSA were acidified through addition of 1/10th volume 10% (v/v) TFA(aq) and 0.5 µL (~15 µg) protein solution was spotted onto a MALDI plate and allowed to air dry. Sample spots were then overlaid with 1 µL of sinapinic acid matrix (10 mg/ml in 50% (v/v) acetonitrile, containing 0.1% (v/v) TFA) and allowed to air dry once more. Spectra were acquired in positive ion linear mode (25 kV accelerating voltage, 250 ns delay

time) over a m/z range of 40000 – 90000 with 50 laser shots per spectrum. External calibration was performed using a BSA standard (AB Sciex).

Neutravidin Capture and Release of RevAmine 4-Modified Hemagglutinin Antigen (HA) Peptide

5 µL of RevAmine **4** (100 mM solution in dry DMSO) was added to 100 µL of 2.25 mM influenza hemagglutinin peptide (Sigma) in 50 mM phosphate buffer, pH 7.4 and the reaction performed for 1 h at RT with occasional mixing. 1 µL of the reaction was analysed by HPLC. Bind-elution was tested using Neutravidin™ agarose (Thermo). Tubes containing 200 µL neutravidin slurry were washed using PBS, pH 7.4 (6 X 100 µL) with pelleting by centrifugation between washes (3000 g, 2 min). RevAmine **4**-modified HA peptide reaction (4.5 µL) in PBS (100 µL) was added to the washed slurry and binding perfomed for 1 h at RT with occasional mixing. A sample (1 µL) of the reaction supernatant was retained for HPLC analysis. The PBS washes (X6) were repeated and then elution performed by addition of 100 mM ammonium bicarbonate, pH 8 (100 µL). The elution solution and beads were mixed thoroughly and then incubated overnight atRT. Next day the slurry was spun (3000 g, 2 min) and the supernatant removed for analysis. An identical sample using non-modified HA peptide was run as a control.

Neutravidin Capture and Release of Biotinylated BSA with PAGE Analysis

To tubes containing 200 µL neutravidin slurry prepared and washed as before was added 3.5 µL of RevAmine 4–modifed BSA (1.5 nmoles) in 50 µL PBS, containing 0.1% Tween 20. Binding was performed for 1 h at RT with occasional mixing. After washing (X6) with PBS, the slurry was divided before the final spin into clean tubes (5 X 40 µL) to test different elution conditions (40 µL eluate). The elution conditions were: 0.1% ammonium hydroxide solution for 1 h at RT; 100 mM ammonium bicarbonate, pH 8.0, 8.5 and 9.0, plus PBS, pH 7.4 overnight at 4°C. The process was repeated using untagged BSA, with three equivalent 40 µL portions of slurry treated with either 100 mM ammonium bicarbonate, pH 9.0 or PBS, pH 7.4 overnight at 4°C and a final sample boiled directly in loading buffer (NuPAGE® LDS, ThermoFisher, UK) without elution. Samples along with ClearPAGE™ molecular weight markers (VWR, UK) were resolved on a 17 well precast 4-12% BisTris SDS-PAGE gel (10 x 10 cm, Expedeon Ltd, UK) in NuPAGE® MOPS running buffer (ThermoFisher, UK) and stained with InstantBlue™ Coomassie (Expedeon Ltd, UK).

Fluorescence Confocal Microscopy of Biotin RevAmine 4-labelled Cells

Jurkat T cells were harvested at approximately 95% confluence, resuspended in Hank's balanced salt solution (HBSS; Lonza) / 1% (v/v) FBS (1 ml) and washed (3 X 1 ml) in this buffer. They were then resuspended in 1 ml phosphate buffered saline (PBS) / 1 mM ethylenediaminetetraacetic acid (EDTA), transferred to low binding microcentrifuge tubes (Sorensen Bioscience) and washed a further three times in PBS/EDTA. After washing, cells were resuspended in PBS at 1×10^7 cells/ml. 10 µL RevAmine **4** (25 µg/µL, reconstituted in dimethyl sulfoxide (DMSO)) was added to 1 ml of cell suspension. 10 µL of DMSO was also added to cells to serve as an unbiotinylated control. Tubes were incubated for 30 minutes at 4°C with end-over-end agitation. Reactions were quenched through addition of 25 µL of 100 mM glycine in PBS with incubation for a further 5 minutes at 4°C with end-over-end agitation. Cells were then spun down, resuspended in 25 mM Tris buffered saline (TBS) and washed three times. One set of biotinylated cells was then incubated in 1 ml of 1% ammonium hydroxide (aq) for 15 min, whilst the others were treated only with TBS. The TBS washes (X3) were then repeated. Biotinylated and control cells were resuspended in fluorescence-confocal microscopy (FCM) buffer (PBS / 0.5% (v/v) BSA / 0.01% (v/v) sodium azide) containing a streptavidin - Alexa Fluor® 568 conjugate (Life Technologies). Cells were incubated at 4°C in the dark for 1 hour, washed three times with FCM buffer and fixed with FCM buffer / 1% (v/v) paraformaldehyde (15 min). 1×10^5 fixed

cells were transferred to microscope slides using a Cytospin for 3 minutes at 1000 rpm and mounted using VECTASHIELD mounting media with 4',6-diamidino-2-phenylindole (DAPI) counterstain (Vector Laboratories). Cells were visualized by immunofluorescence using a Leica TCS SP2 UV laser-scanning confocal microscope set up to detect tetramethyl rhodamine isothiocyanate (TRITC) (excitation 547 nm, emission 572 nm) and DAPI (excitation 345 nm, emission 455 nm). Baseline TRITC emission levels were set using unlabelled cells and all labelled cells imaged using these settings.

RevAmine 7 Protein Modification, Neutravidin Capture and Release

Stock aqueous solutions (15 μM) were prepared of *Bacillus licheniformis* nuclease B (Dr L. Hewitt, ICaMB, Newcastle), *Bos taurus* cytochrome C (Sigma, UK) and *Equus caballus* apomyoglobin (Sigma, UK). A 5 μL aliquot of stock **7** (100 mM in dry DMSO) was diluted X10 using dry DMSO to produce a working solution at 10 mM. Stock protein (100 μL, 1.5 nmoles) was mixed with 33.3 μL of 200 mM sodium phosphate buffer, pH 7.4 (for final concentration of 50 mM) to which 1.5 μL of 10 mM **7** was added (X10 fold excess over protein). After incubating the reaction for 1h at RT, the modified protein mixture was desalted into water using 0.5 mL, 7K MWCO Zeba™ spin columns (Thermo, UK) to remove excess reagent prior to mixing with Neutravidin™-Agarose beads (Pierce). Briefly, 300 μL neutravidin slurry was placed in a 2 mL glass tube (VWR, UK) and prepared by centrifuging (3000 g, 1 minute) to remove storage buffer. The beads were then washed three times with 50 mM sodium phosphate buffer, pH 7.4 (300 μL) and the supernatant discarded. Next 100 μL of phosphate buffer was added to the pelleted resin followed by half of the desalted 7-modified protein sample (~60 μL) with gentle mixing. Neutravidin capture was allowed to proceed for 30 min at RT. Following another spin the supernatant was removed (for LC-MS confirmation of capture) and the resin washed three times as before with phosphate buffer, followed by 4 X 300 μL washes with water. Elution was initiated by addition of a fresh solution of 200 mM ammonium bicarbonate buffer, pH 8, containing 20 mM DTT (300 μL) with gentle mixing. Following overnight incubation at RT (14 h), a fresh 300 μL aliquot of ammonium bicarbontate / DTT was added to the reaction mix and the elution reaction continuted for a further 10 h before the supernatant was harvested by centrifugation (3000 g, 2 minute).

Trapping LC-MS Analysis

Protein eluate from the neutravidin capture and release experiment described above (~500 μL) was acidified on ice by addition of 10% formic acid (aq) in 3 X 50 μL aliquots with gentle mixing and allowing the effervescence to subside. The acidified protein mixture was injected onto a 'Cap Trap' column (nanoLCMS Solutions, CA, USA) and elution performed using an 1100 HPLC system (Agilent, UK) at a flow rate of 0.05 mL/min. The initial conditions were 5% buffer B in buffer A and the standard gradient conditions were 5-70% buffer B in buffer A over 22 min. A final pulse to 100% B over 5 min followed by an immediate return to 5% B completed the elution gradient. Buffer A: 0.1% formic acid in water; Buffer B: 0.1% formic acid in MeCN. Electrospray data was acquired throughout using an LTQ-FT mass spectrometer (Thermo) with a FT-MS resolution setting of 100,000 at *m/z* = 400 and an injection target value of 1,000,000. LC-MS spectral data was inspected using Qualbrowser software (Thermo) and FT spectral deconvolutions of protein charge state distributions were performed using the built-in Xtract™ software module. Calculated molecular weights were determined from the amino acid sequence for NucB (UniProtKB - F1BV52) and apomyoglobin (UniProtKB - P68082) using the ExPASy ProtParam tool (http://web.expasy.org/protparam/?_ga=1.249982593.600072476.1489489500), whilst that of cytochrome C was obtained via PDB code 2B4Z (http://www.rcsb.org/pdb/explore.do?structureId=2b4z) contained within Liu and Konermann (2009)[1].

***E.coli* Surface Labelling Experiment**

Batches of cells (3 per treatment) were prepared by spinning down 1 X 10^9 – 1 X 10^{10} of *E.coli* cells (*amiABC*)[2] and then carefully washing each cell pellet with PBS (3 X 1 ml). One set of triplicate cell pellets were held back for labelling until after cell lysis to provide a subtraction dataset. Labelling was performed by resuspending the pellets in 1ml PBS to which was added 10 µL 0.5M stock RevAmine **7** in DMSO. The cells plus reagent were then gently rocked at 4°C for 1h. The biotinylation reaction was quenched by briefly washing the cells with 0.5M glycine in PBS (3 X 1ml) followed by pelleting. Cell pellets were lysed by brief sonication in 0.5 ml RIPA buffer (10 mM phosphate buffer, pH 7.2, 150 mM NaCl, 1% Triton X-100, 0.5% sodium deoxycholate, 0.1% SDS, plus 1 mM EDTA) containing containing 1:100 (v/v) protease inhibitor mix (Halt™, Thermo). Cells were kept on ice and pulsed briefly (10 - 20s) followed by cooling on ice for 30 s (3 repeats). To complete this process the lysates were incubated on ice for a further 30 min with occasional gentle vortexing. Lysates from the unlabelled cell pellets set-aside earlier were then biotin labelled using RevAmine **7** via addition of 10 µL of 0.5M stock reagent and allowing the reaction to proceed for 1 h at 4°C. A quench was performed by addition of 20 µL of 0.5 M glycine in PBS. All lysates were then cleared via centrifugation (16,000g, 15 min, 4°C) and the supernatant collected. Glycerol was added to a final concentration of 10% to help prevent protein aggregation and the samples were then stored at -20°C until required.

Biotinylated proteins were enriched from lysates using Neutravidin™-Agarose beads (Pierce). Briefly, bead slurry (400 µL) was pipetted into tubes and prepared by spinning (3000g, 2 min) to pellet, followed by washing (400 µL, X3) using an equal volume of RIPA buffer : PBS (1:1). The beads were then incubated with biotinylated cell lysate for 2h at 4°C with occasional mixing. The beads were then pelleted and the supernatant removed, washed X3 with diluted RIPA buffer as above to remove unbound proteins and finally with PBS (400 µL, X3) to remove non-ionic detergent. Biotinylated protein material was released via incubation with 100 mM ammonium bicarbonate, pH 9.0 (overnight, 4°C), containing 0.2% ProteaseMax surfactant (Promega).

After spinning (3000g, 5 min) to pellet the agarose beads, the supernatant was transferred to a clean tube, the beads were washed a further two times with the same buffer, spun (3000g, 2 min) and the supernatants combined and dried *in vacuo*.

Dried samples were reconstitued in 10 µL denaturing, reducing buffer (0.5M Tris, pH 8, containing 6M guanidine, 10 mM TCEP and 50% acetonitrile (v/v)). Samples were then alkylated in the dark by addition of 2 µL, 100mM iodoacetamide (15 min, RT).

After alkylation, 85 µL of 50 mM ammonium bicarbonate, pH 8 buffer was added, followed by trypsin at 1:50 enzyme:substrate ratio. Digestion was performed overnight at 35 °C.

Next day the samples were acidified by addition of 1/10th volume 10% trifluoroacetic acid (aq) and desalted (StageTip C-18, Thermo) into sample vials, dried *in vacuo* and stored at -20°C until nano LC-MS/MS analysis.

Nanoelectrospray MS/MS Analysis

Dried desalted peptide digests were resuspended in 0.1% formic acid (aq) and injected onto a 5 X 0.3 mm Zorbax 300SB-C18 trapping column (Agilent, UK) in-line with a 75 µm X 100 mm BEH130 C18 capillary column (Waters, UK) running on a NanoAcquity UPLC system (Waters, UK). The gradient conditions were 5-35% buffer B in buffer A over 120 min at a flow rate of 0.4 µL/min. Buffer A: 0.1% formic acid in water; Buffer B: 0.1% formic acid in acetonitrile.

Nanoelectrospray data was acquired using an LTQ-FT mass spectrometer (Thermo). Survey MS scans were performed over the mass range m/z = 300 – 1500 in data-dependent mode. Data was acquired with a FT-MS resolution setting of 100,000 at m/z = 400 and a Penning trap injection target value of 1,000,000. The top five ions in the survey scan were automatically subject to collision-induced dissociation MS/MS in the linear ion trap region of the instrument at an injection target value of 100,000, using a normalized collision energy of 30% and an activation time of 30 ms (activation Q = 0.25). Precursor ion charge state screening was enabled and only doubly- and triply-charged ions were selected. Dynamic exclusion was enabled

and precursor masses were selected for MS/MS with a repeat count of 2 (within 15 s), then excluded from further selection for a duration of 30 s.

Proteomic Data Analysis

All LC-MS/MS data files from analysis of digest peptides were searched using the Mascot™ MS/MS ion search tool (Matrix Science, London) with the following search parameters; enzyme: trypsin, fixed modifications: carbamidomethyl (C), variable modifications: pyro-Glu (N-term Q) and oxidation (M), peptide mass tolerance: ± 10 ppm, fragment mass tolerance: ± 0.6 Da, max missed cleavages: 3. In the *E.coli* surface labelling experiment searches were performed against an in-house *E.coli* sequence database downloaded from UniProt (dated 20140711, 4375 sequences, 1400643 residues). A PANTHER classification system search (http://pantherdb.org/)[3] was performed using a list of unique surface-related proteins generated by subtracting the proteins identified in the post-lysis labelling Mascot search from those identified in the surface labelling Mascot search (supplementary data ST1 – ST3). The analysis performed was a PANTHER overrepresentation test (release 20150430) and the annotation version and release date were : GO Ontology database, released 2015-08-06. The Mascot-returned protein accession codes were translated into gene names using the DAVID Gene Accession Conversion Tool at LHRI (Leidos Biomedical Research, Inc., USA, https://david.ncifcrf.gov/content.jsp?file=conversion.html)[4] . The gene list was uploaded and searched against a reference E.coli gene list targeting the annotation data set *'GO cellular component complete'*. The analysis summary report for this search is reproduced in supplementary figure S14.

[1] J. Liu and L. Konermann, *J Am Soc Mass Spectrom.* **2009** 20, 819-828.

[2] C. Heidrich, A. Ursinus, J. Berger, H. Schwarz, J.V. Höltje, *J Bacteriol.* **2002** 184, 6093-6099.

[3] H. Mi, A. Muruganujan and P. D. Thomas, *Nucl. Acids Res,* **2013,** 41, D377-386.

[4] D.W Huang, B.T. Sherman, R.A. Lempicki, *Nat Protoc*, **2009**, 4, 44-57.
